# Thermodynamic and Structural Modelling of Non-Stoichiometric *Ln*-Doped UO_2_ Solid Solutions*,* *Ln* = {La, Pr, Nd, Gd}

**DOI:** 10.3389/fchem.2021.705024

**Published:** 2021-11-08

**Authors:** Victor L. Vinograd, Andrey A. Bukaemskiy, Giuseppe Modolo, Guido Deissmann, Dirk Bosbach

**Affiliations:** Institute of Energy and Climate Research IEK 6, Nuclear Waste Management and Reactor Safety, Forschungszentrum Jülich, Jülich, Germany

**Keywords:** UO_2_ fluorite, non-stoichiometry, oxidation resistance, lattice parameter, thermodynamic modelling

## Abstract

Available data on the dependence of the equilibrium chemical potential of oxygen on degrees of doping, *z*, and non-stoichiometry, *x*, *y*, in U_1-*z*_ *Ln*_*z*_O_2+0.5(*x*-*y*)_ fluorite solid solutions and data on the dependence of the lattice parameter, *a*, on the same variables are combined within a unified structural-thermodynamic model. The thermodynamic model fits experimental isotherms of the oxygen potential under the assumptions of a non-ideal mixing of the endmembers, UO_2_, UO_2.5_, UO_1.5_, *Ln*O_1.5_, and *Ln*_0.5_U_0.5_O_2_, and of a significant reduction in the configurational entropy arising from short-range ordering (SRO) within cation-anion distributions. The structural model further investigates the SRO in terms of constraints on admissible values of cation coordination numbers and, building on these constraints, fits the lattice parameter as a function of *z*, *y,* and *x*. Linking together the thermodynamic and structural models allows predicting the lattice parameter as a function of *z*, *T* and the oxygen partial pressure. The model elucidates contrasting structural and thermodynamic changes due to the doping with LaO_1.5_, on the one hand, and with NdO_1.5_ and GdO_1.5_, on the other hand. An increased oxidation resistance in the case of Gd and Nd is attributed to strain effects caused by the lattice contraction due to the doping and to an increased thermodynamic cost of a further contraction required by the oxidation.

## 1 Introduction

One important aspect of a safety case for a geological repository for the high-level nuclear waste (e.g., UO_2_-based spent nuclear fuel, SNF) is to characterize the corrosion behaviour of and the consequent radionuclide release from the disposed wastes, after waste containers will be eventually breached due to corrosion. After unloading from a reactor (e.g., a light water reactor), the SNF still contains ∼95 wt% of uranium and ∼5 wt% of fission products and transuranium elements (TRU), Pu, Am, and Cm. Fission and activation products formed during irradiation of oxide fuels (e.g., UO_2_) could be classified into three categories, namely: 1) those forming metallic inclusions (e.g., Mo, Tc, Ru, Rh, and Pd), 2) those forming oxide precipitates (e.g., Rb, Cs, Ba, Zr, Nb, and Mo), and 3) those remaining as components of a solid solution with UO_2_ (e.g., Sr, Zr, Nb, lanthanides (Ln), and TRU) (Kleykamp, 1988; Bruno and Ewing, 2006; Carbol et al., 2012; Ewing, 2015).

In the repository system groundwater is the principal medium for the transport of radionuclides from the waste to the environment including corrosion of the waste. The unique chemical conditions are characterized by 1) generally reducing conditions due to a significant amount of hydrogen produced due to the anoxic corrosion of metallic waste containers, 2) the presence of locally formed oxidizing conditions caused by radiolytic products such as H_2_O_2_, and 3) complex groundwater chemistry. The radiolytic oxidants evoke locally oxidizing effects at the spent fuel surface leading to an oxidative dissolution of the waste, due to the oxidation of poorly soluble U(IV) to easily soluble U(VI) (Shoesmith, 2000; Eriksen et al., 2012; Shoesmith et al., 2013; Bosbach et al., 2020; Hansson et al., 2021).

During the last decades, a number of studies addressed the corrosion behaviour of SNF, leading to a good phenomenological understanding of the instant release of radionuclides and long-term corrosion rates (Kleykamp, 1985; Fanghänel et al., 2013; Ewing, 2015; Lemmens et al., 2017; Curti and Kulik, 2020). Here we focus on the question how the doping of uranium dioxide by a variety of fission and activation products affects the corrosion rate under repository conditions.

As fission yields of lanthanides, such as La, Ce, Pr, and Nd, due to the fission of ^235^U and ^239^Pu are high (Kleykamp, 1985), leading to significant amount of lanthanides dissolved in the UO_2_ matrix (up to 2–4 at%), UO_2_–*Ln*O_1.5_ systems may serve as analogues of spent fuels illustrating certain aspects of burn-up on chemical stability (Ferry et al., 2005; Bruno and Ewing, 2006; Carbol et al., 2012). Effects of oxidative dissolution have been recently measured in aqueous (typically with H_2_O_2_ added) and in electrochemical systems both at corrosive (rest) potentials and at potentials promoting the conversion of U^+4^ to higher oxidation states. Such experiments applied to Gd-, Dy-, and Y-bearing samples have shown that the doping reduces dissolution yields and oxidative (anodic) currents relative to those measured for pure UO_2_ (Trummer et al., 2010; Razdan and Shoesmith, 2013; Casella et al., 2016; Kim et al., 2017; Liu et al., 2017; Barreiro Fidalgo and Jonsson, 2019). Similar reductions of oxidation rates have been measured for simulated fuels (SIMFUELs) that in addition to lanthanides contain a variety of other dopants (He et al., 2007; Nilsson and Jonsson, 2011; Razdan and Shoesmith, 2013; Liu et al., 2017).

Despite this effort, mechanisms of resistance to oxidation in *Ln*- or Y-doped systems and in chemically more complex simulated fuels remain unclear. In several studies the stabilizing effect was linked to the formation of *Ln*-V_O_ clusters (V_O_ denotes an oxygen vacancy), which are thought to reduce the number of vacant sites that could host oxygen anions (Razdan and Shoesmith, 2013; Kim et al., 2017; Liu et al., 2017). The formation of these clusters has been discussed in the frame of a point-defect model of *Ln*-doped UO_2_ by Park and Olander (Park and Olander, 1992). However, the fraction of such clusters in hyper-stoichiometric (oxidized) samples is predicted to be very small. Casella et al. (Casella et al., 2016) suggested that the stabilizing effect of *Ln* ^3+^ could be related to its effectively negative charge that repels O-interstitials, O_i_, and thus limits the fraction of interstitial sites able to accommodate an excess oxygen. Kim et al. (Kim et al., 2001) proposed that the decreased rates of oxidation in air measured on Gd-doped samples were due to a decreased fraction of U^+4^ (the fraction of cations able to be oxidized) caused by the ingrowth of Gd^+3^ and U^+5^. Furthermore, as most of the experiments have been done with dopants that promoted lattice contraction, a proposition was made that the stabilizing effect could be caused by a reduced rate of diffusion of oxygen anions into the interstitial sites promoted by the contraction (Kim et al., 2017). The study of Kim et al. (Kim et al., 2017) further proposed that variations in electrochemical reactivity of Gd-doped samples characterized by different types of non-stoichiometry could be related to different degrees of lattice contraction observed on Gd-doping in hypo-, hyper- and strictly-stochiometric samples. The largest effect of Gd on the oxidative anodic currents was noted in hyper-stoichiometric samples, those characterised by the strongest decrease in the lattice parameter with doping (Kim et al., 2017).

The similarity of stabilizing effects due to the doping observed in electrochemical oxidation (He et al., 2007; Razdan and Shoesmith, 2013; Kim et al., 2017; Liu et al., 2017), in oxidative dissolution (Trummer et al., 2010; Casella et al., 2016; Barreiro Fidalgo and Jonsson, 2019) and in air oxidation experiments (Kim et al., 2001) suggests that all these phenomena might be linked to a common thermodynamic factor.

The resistance of the UO_2±*δ*_ solid solution to oxidation in air, where *δ* denotes deviation from stoichiometry, can be equated to the thermodynamic force (free energy) required for an insertion of an extra oxygen anion into its structure. This force at the thermodynamic equilibrium is proportional to the chemical potential of oxygen gas that is required for attaining a certain degree of hypo- or hyper-stoichiometry. Indeed, a large experimental effort has been invested in studying effects of *Ln*-doping on the chemical potential of oxygen in equilibrium with a given degree of hypo- or hyper-stoichiometry at a fixed temperature (Hagemark and Broli, 1967; Tetenbaum and Hunt, 1968; Javed, 1972; Saito, 1974; Une and Oguma, 1983a; Nakamura and Fujino, 1987; Lindemer and Sutton, 1988; Yoshida et al., 2011; Lee et al., 2016a; McMurray and Silva, 2016). These experiments invariably show that the larger the doping, the higher is the chemical potential, or, equivalently, the partial pressure of oxygen, at which a given degree of non-stoichiometry can be attained. Studies based on point-defect theory (Park and Olander, 1992), CALPHAD methodology (Saunders and Miodownik, 1998; Hillert, 2001) and on dual solution Gibbs energy minimization method (Karpov et al., 2001; Kulik et al., 2013) provided thermodynamically sound models able to predict the equilibrium partial pressure of oxygen at a given degree of doping and/or non-stoichiometry (Guéneau et al., 2011; McMurray et al., 2013; Degueldre et al., 2014; McMurray et al., 2015; Lee et al., 2016a; McMurray and Silva, 2016; Curti and Kulik, 2020). However, little effort has been so far invested in correlating these models with lattice parameter data. We argue here that a further understanding of mechanisms of oxidation resistance could be gained from a model that provides an accurate thermodynamic description of data on the dependence of the oxidation potential on the dopant type, on the degree of doping and non-stoichiometry, and simultaneously evaluates the dependence of the lattice parameter of UO_2_ on the same set of parameters. Previous research has shown that the lattice parameter, *a*, of fluorite solid solution changes markedly as a function of the type and degree of doping and the extent of non-stoichiometry (Ohmichi et al., 1981; Fukushima et al., 1983; Schreinemachers et al., 2014; Lee et al., 2016b; Schreinemachers et al., 2020). Combining this abundant structural information with thermodynamic data may provide means for understanding the role of the structure in the oxidation resistance of doped UO_2_.

Here we develop structural-thermodynamic models for U_1-*z*_ *Ln*_*z*_O_2+0.5(*x*-*y*)_ solid solutions (*Ln* = {La, Pr, Nd, Gd}), in which the non-stoichiometry, 0.5(*x*-*y*), originates due to the presence of a fraction *x* of UO_2.5_ (U = U^+5^) and/or a fraction *y* of AO_1.5_ (A = *Ln* ^+3^, U^+3^) components. The aim is to predict the non-stoichiometry, the equilibrium cation species fractions and the lattice parameter, *a*, as functions of the composition, *z*, the temperature *T*, and the partial pressure of oxygen, $P_{{\text{O}}_{2}}$ , that is measured relative to the standard pressure, $P^{0}$ ( $P^{0}=101325\text{ Pa}$ ). The parameters *x* and *y* thus define the degrees of hyper- and hypo-stoichiometry reflecting the presence of oxygen interstitials or oxygen vacancies, respectively. The term “cation species”, in contract to simply a “cation”, includes the information on both the chemical type of a cation and its coordination number in the fluorite solid solution. For example, U^4,7^ denotes U^+4^ in the 7-fold coordination. The coordination numbers are important as they determine the cation sizes and, thus, the lattice parameter. The systems of *Ln* = {La, Pr, Nd, Gd} are particularly convenient for such a study as they cover contrasting cases of the lattice response to *Ln*O_1.5_ doping: the extension, i.e. a positive d*a*/d*x* slope (La), a close to zero slope (Pr) and a negative slope (Nd, Gd). The thermodynamic part is worked out via a fit to $\log\left(P_{{\text{O}}_{2}}/P^{0}\right)$ data vs. non-stoichiometry data at a fixed value of *T*. The structural part is based on fitting the lattice parameter data via an ion-close-packing concept (Ohmichi et al., 1981; Lee et al., 2016b; Bukaemskiy et al., 2021). This concept includes the dependence of average cation radii on average cation coordination numbers that vary as functions of composition and non-stoichiometry and assigns a certain fixed radius to the oxygen vacancy (Bukaemskiy et al., 2021; Vinograd and Bukaemskiy, 2021). The cation radii are either taken from Shannon (Shannon, 1976) or fitted to available *a* vs. *z*, and *a* vs. O/M data. The relationship between the thermodynamic and structural description is set via common assumptions on cation and anion distribution and ordering. These assumptions, on the one hand, constrain equations for the entropy/enthalpy of mixing and, on the other hand, limit allowed values of cation coordination numbers imposing constrains on the average cation radius and, thus, on the lattice parameter.

An important practical aspect of the present study is the development of a model that relates the equilibrium lattice parameter, *a*, to *z*, *x*, and *y* and simultaneously to *T* and $P_{{\text{O}}_{2}}$ . The model can, thus, be used to calculate $P_{{\text{O}}_{2}}$ or *T* at synthesis conditions from *a* vs. *z* plots and to predict $P_{{\text{O}}_{2}}$ and *T* that are required for synthesising samples with a desired degree on non-stoichiometry and a desired derivative d*a*/d*x*. The model consistently reproduces the relationship that the higher the level of doping, the higher is the oxidation potential that is required to equilibrate a sample of a given degree of hyper-stoichiometry at a given temperature, while this dependence is enhanced within the row of La < Pr < Nd < Gd.

## 2 Methods and Models

### 2.1 Thermodynamic Model

**FIGURE 1:**
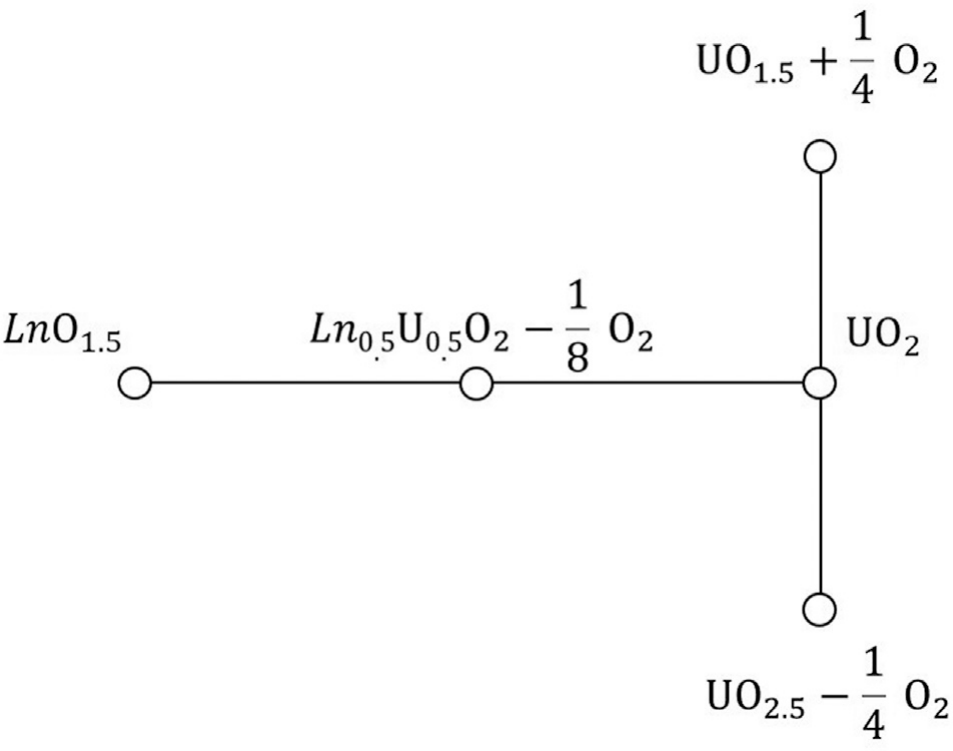
Relationship between endmembers of the thermodynamic model. Note, that all Ln-free endmembers have the same chemical composition of UO_2_.

The thermodynamic model assumes a system of a constant chemical composition at a given temperature. The composition of the solid phase is defined by the fractions *z* and 1–*z* of the chemical endmembers with the compositions of *Ln*O_1.5_ and UO_2_, respectively. We also assume that a system includes a large fixed amount of O_2_ gas with its partial pressure maintained at a given value. The endmember UO_2_ is assumed to exist in three different structural forms, namely, the stoichiometric UO_2_, the hypo-stoichiometric UO_1.5_ associated with ¼ moles of O_2_ gas and the hyper-stoichiometric UO_2.5_ associated with a negative amount of ¼ moles of O_2_. The negative sign implies that when excess O is inserted into UO_2_, an equivalent quantity (in moles) of O_2_ gas annihilates in the system. The equal sum of the endmembers *Ln*O_1.5_ and UO_2_ reacts producing the fifth endmember, *Ln*_0.5_U_0.5_O_2_–1/8 O_2_ (Figure 1), which is also associated with a negative amount of O_2_ gas. When not explicitly indicated, the list of the endmembers will be associated with low case indices in the order 1- UO_1.5_, 2- UO_2_, 3- UO_2.5_, 4- *Ln*O_1.5_, and 5- *Ln*_0.5_U_0.5_O_2_. This set of endmembers is sufficient to describe variations in stoichiometry of fluorite solid solutions at not too large fractions of *Ln*O_1.5_. Fractions larger than *z* = 0.5 may require the consideration of the U_1/3_ *Ln*_2/3_O_2_ endmember to describe stoichiometric states and the UO_3_ endmember to describe hyper-stoichiometry. Both these endmembers contain U^+6^. As the final aim of this study is to characterize the oxidation resistance of SNF, in which the content of *Ln*O_1.5_ does not exceed few atomic percent, we have chosen to limit the set of U species to U^+3^, U^+4^, and U^+5^. Thus, the present model should be applied with caution when *z* > 0.5. The gas phase component associated with an endmember will be often omitted for brevity. For example, the endmember UO_2.5_ means a UO_2.5_ coexisting with a negative amount of ¼ moles of O_2_ gas. This recognition is important in the following definition of the standard Gibbs free energies of the endmembers. The free energy of each endmember is composed of two contributions, one arising from a solid-state transformation (insertion/deletion of 0.5 mole of O into/from the UO_2_ structure) and one arising from a creation (or an annihilation) of an equivalent amount of O_2_ gas. The latter contribution is proportional to the chemical potential of O_2_. The sum of these contributions is modelled relative to an equivalent combination of the free energies of *Ln*O_1.5_ and stoichiometric UO_2_. The free energies of the latter endmembers are set equal zero. As the standard free energies of the other endmembers explicitly depend on the chemical potential, a change in the chemical potential, or a change in the partial pressure of O_2_, induces a change in endmember fractions. When the pressure is low (the chemical potential of O_2_ is strongly negative), UO_1.5_ + ¼ O_2_ endmember is stabilised over UO_2_. When the chemical potential is close to zero, UO_2.5_ – ¼ O_2_ is stabilised over UO_2_, consistently with the thermodynamic instability of UO_2_ in air. A shift of the endmember fractions towards the formation of the UO_2.5_ and *Ln*_0.5_U_0.5_O_2_ implies an oxidation of a fraction of U^+4^ to U^+5^, while a relative increase in the fraction of UO_1.5_ implies a reduction of a fraction of U^+4^ to U^+3^. The equilibrium endmember fractions are obtained via the minimization of the Gibbs free energy, which includes an additive sum of endmember contributions, a non-ideal term arising from the interactions between the endmembers and an entropic term. The equilibrium calculations are done here using a specially written FORTRAN code.

#### 2.1.1 A Model of UO_2+0.5(*x-y*)_

Pure UO_2_ fluorite is described here as a combination of two binary solid solution models spanning between the endmembers UO_2_ and UO_1.5_, in the first case, and UO_2_ and UO_2.5_, in the second case. U^+6^ states, i.e. the UO_3_ endmember, are not included within the present effort. The free energy of stoichiometric UO_2_ is set equal to zero, while the free energies of UO_2.5_ – ¼ O_2_ and UO_1.5_ + ¼ O_2_ endmembers are set equal to the effects of the reactions

$$\;{\text{UO}}_{2}={\text{UO}}_{2.5}-\frac{1}{4}{\text{O}}_{2}$$ (1)

and

$${\text{UO}}_{2}={\text{UO}}_{1.5}+\frac{1}{4}{\text{O}}_{2}$$ (2)

For example, the effect of Eq. 1 splits into the effect of addition of 0.5 mole of O into the solid and the effect of annihilation of ¼ moles of O_2_ gas prepared at a given temperature *T* with a given partial pressure

$$G_{\text{UO}2.5}=\Delta G_{\text{UO}2.5}^{0}-\left(T-T^{0}\right)\Delta S_{\text{UO}2.5}^{0}-\frac{1}{4}\mu_{{\text{O}}_{2}}^{T,P^{0}}$$ (3)

Similarly, the effect of Eq. 2 splits into the effect of removing of 0.5 mole of O from the solid and the effect of creating of ¼ moles of O_2_ gas

$$G_{\text{UO}1.5}=\Delta G_{\text{UO}1.5}^{0}-\left(T-T^{0}\right)\Delta S_{\text{UO}1.5}^{0}+\frac{1}{4}\mu_{{\text{O}}_{2}}^{T,P^{0}}$$ (4)

The parameters $\Delta G_{i}^{0}$ and $\Delta S_{i}^{0}\;$ are determined by fitting. The chemical potential of O_2_ gas is computed as

$$\mu_{{\text{O}}_{2}}^{T,P^{0}}=-S_{{\text{O}}_{2}}^{0}\left(T-T^{0}\right)+C_{p_{{\text{O}}_{2}}^{0}}\left(T-T^{0}-Tln\left(\frac{T}{T^{0}}\right)\right)+RT\ln\left(P_{{\text{O}}_{2}}/P^{0}\right)\;$$ (5)

where $S_{{\text{O}}_{2}}^{0}$ = 205.1373 J/K/mol and $C_{p_{{\text{O}}_{2}}^{0}}$ = 29.355 J/K/mol and where $P^{0}$ = 101,325 Pa, $T^{0}=298.15k$ (Finnis et al., 2005).

The reference Gibbs free energies for hypo-stoichiometric, UO_2-0.5*y*_, and hyper-stoichiometric solutions, UO_2+0.5*x* **,**_ are written as follows

$$G_{\text{hypo}}=yG_{\text{UO}1.5}+\left(1-y\right)G_{\text{UO}2}$$ (6)

and

$$G_{\text{hyper}}=xG_{\text{UO}2.5}+\left(1-x\right)G_{\text{UO}2}$$ (7)

respectively, where *y* and *x* are the mole fractions of the components UO_1.5_ and UO_2.5_. Naturally, only the solid part of an endmember contributes to the structural formula, however, an equivalent positive or negative mole fraction of O_2_ gas is always associated with it. Thus, the quantities in Eqs. 6, 7 depend on the chemical potential of O_2_. The excess free energies are given as follows

$$G_{\text{hypo}}^{\text{exess}}=y\left(1-y\right)\left(W_{13}^{\text{h}}-TW_{13}^{\text{s}}\right)$$ (8)

and

$$G_{\text{hyper}}^{\text{exess}}=x\left(1-x\right)\left(W_{23}^{\text{h}}-TW_{23}^{\text{s}}\right)$$ (9)

where $W_{13}^{\text{h}}\;\text{and}\;W_{13}^{\text{s}}$ refer to the interaction between solid UO_1.5_ and UO_2_ and $W_{23}^{\text{h}}\;\text{and}\;W_{23}^{\text{s}}$ refer the interaction between solid UO_2_ and UO_2.5_. The upper indices “h” and “s” denote enthalpic and entropic parts of the interaction parameters. All these parameters are determined by fitting.

Modelling the configurational entropy of anions requires consideration of effects of local order. Following (Bukaemskiy et al., 2021; Vinograd and Bukaemskiy, 2021) we assume a type of a short-range ordering in which two vacancies cannot approach each other closer than the fourth nearest anion-anion distance. Such a distribution can be emulated by restricting the location of vacancies to a simple cubic sublattice of the anion lattice in which the nearest distance is equal to the fourth nearest distance of the original anion lattice. This model implies that the mixing of vacancies and oxygen anions occurs on 1/8-th of available anion sites, while the concentration of vacancies within the sublattice is 8 times larger than the average concentration of vacancies of 0.25*y*. These assumptions lead to the equation

$$S_{\text{O}/\text{V}}^{\text{conf}}=-0.25R\left(2y\ln\left(2y\right)+\left(1-2y\right)\ln\left(1-2y\right)\right)$$ (10)

which is valid for 0 < *y* < 0.5. A more detailed derivation of Eq. 10 and of other entropy equations that appear further in the text is given in the Supplementary Materials.

The hyper-stoichiometric solution is characterized by the presence of oxygen interstitials, which are assumed to occupy octahedral sites. One of recent simulation studies provided arguments in favour of a model containing mono- and di-interstitials, with the proportion of di-interstitials increased at larger values of *x* (Palomares et al., 2019). Two types of di-interstitial cluster, the Willis cluster (Willis, 1978) and the split di-interstitial (Andersson et al., 2009) have been proposed based on neutron diffraction experiments and ab initio calculations. On the other hand, structural studies of compositions close to U_4_O_9_ stoichiometry (*x* = 0.5) suggested the presence of cubooctahedral clusters composed of interstitials and lattice anions shifted from their ideal positions (Bevan et al., 1986). We assume here that the appearance of cubooctahedral clusters signifies a transition from a defect fluorite type solid solution to a solid solution based of a U_4_O_9_-type structure and do not make an attempt of modelling the latter. We assume that the distribution of interstitials in hyper-stoichiometric defect fluorite could be simulated assuming the presence of mono- and di-interstitials only. To emulate the presence of just two types of clusters (i.e., the absence of higher-order clusters) we assume the existence of a sublattice within the ideal FCC lattice of interstitials, which is composed of an ordered arrangement of isolated (non-overlapping) pairs of initially vacant interstitial sites. Such an arrangement implies that only ¼ of totally available vacant sites can be occupied by O-interstitials. The concentration of interstitials within such a sublattice increases in four times from the value of 0.5*x* to the value of 2*x*. We further assume that the occupation of pairs of vacant sites within the sublattice occurs randomly. These assumptions lead to the following equation for the entropy of mixing

$$S_{\text{Oi}/\text{Vi}}^{\text{conf}}=-0.25R\left(2x\ln\left(2x\right)+\left(1-2x\right)\ln\left(1-2x\right)\right)$$ (11)

which is valid in hyper-stoichiometric domain of 0 < *x* < 0.5. Accidentally, this equation is similar to Eq. 10. This circumstance appears important as the thermodynamic data on UO_2+0.5(*x-y*)_ show a nearly symmetric relationship between hypo- and hyper-stoichiometric branches of $\log\left(P_{{\text{O}}_{2}}/P^{0}\right)$ data vs. non-stoichiometry.

The splitting of the model into the independent “hypo” and “hyper” domains is based on the assumptions that the reactions

$$\text{O}={\text{O}}_{\text{i}}+\text{V}$$ (12)

and

$$2{\text{U}}^{+4}={\text{U}}^{+3}+{\text{U}}^{+5}$$ (13)

do not occur separately from each other and that both are strongly shifted to the left. Practically, we assume that oxygen vacancies and oxygen interstitials annihilate each other, and the same assumption is made for U^+3^ and U^+5^ cations. These assumptions are reasonable at not too high temperatures considering large enthalpy effects of ∼4 and ∼1.5 eV computed for these reactions ab initio (Nerikar et al., 2009; Cooper et al., 2018). The present model is thus not intended for modelling of UO_2_ at very high temperatures (∼2000 K and above), where these assumptions become invalid. A consequence of these assumptions is that the chemical potential of O_2_ in equilibrium with pure UO_2_ cannot be defined, i.e. pure UO_2_ coexists with a range of $\log\left(P_{{\text{O}}_{2}}/P^{0}\right)$ values. A further simplification made here is that the entropy effects associated with U^+4^/U^+5^ and U^+4^/U^+3^ mixing cannot be separated out from the effects of O_i_/V_i_ and O/V mixing. Thus, we count only the entropy effects associated with the anions. A possible physical interpretation behind ignoring the entropy effects due to U^+4^/U^+5^ and U^+4^/U^+3^ mixing is that the electron distribution that distinguishes U^+5^/U^+3^ from U^+4^ adjusts itself spontaneously to a given arrangement of oxygen vacancies or oxygen interstitials.

#### 2.1.2 *Ln*-Doped UO_2_-Based Solid Solutions

*Ln* ^+3^ can be charge balanced in the fluorite solid solution according to two different schemes

$${\text{U}}^{+4}+\;\frac{1}{2}{\text{O}}^{-2}=Ln^{+3}+\frac{1}{2}\text{V}$$ (14)

and

$$2{\text{U}}^{+4}=Ln^{+3}+{\text{U}}^{+5}\;$$ (15)

The first scheme implies the creation of oxygen vacancies. The second scheme requires an oxidation of one U^+4^ cation per one *Ln* ^+3^. These schemes are put into the correspondence with *Ln*O_1.5_ and *Ln*_0.5_U_0.5_O_2_ endmembers, respectively, where the latter implies the simultaneous addition of equal fractions of *Ln* ^+3^ and U^+5^. The free energy of *Ln*_0.5_U_0.5_O_2_ endmember is defined here via the reaction

$$\frac{1}{2}{\text{UO}}_{2}+\frac{1}{2}Ln{\text{O}}_{1.5}=Ln_{0.5}{\text{U}}_{0.5}{\text{O}}_{2}-\frac{1}{8}\;{\text{O}}_{2}$$ (16)

as follows

$$G_{Ln0.5\text{U}0.5\text{O}2}=\Delta G_{Ln0.5\text{U}0.5\text{O}2}^{0}-\left(T-T^{0}\right)\Delta S_{Ln0.5\text{U}0.5\text{O}2}^{0}-\frac{1}{8}\mu_{{\text{O}}_{2}}^{T,P^{0}}$$ (17)

According to the previously made assumption, U^+5^ cannot occur together with U^+3^, as these cations annihilate each other via inverse reaction (Eq. 13). Thus, the *Ln*_0.5_U_0.5_O_2_ and UO_1.5_ endmembers cannot occur together. Due to this circumstance, doped hypo-stoichiometric solid solutions at a given fraction *z* of *Ln*O_1.5_ could be conveniently split into two types, I and II, defined by the inequalities 0.5 > *y* > *z* and *z* > *y* > 0, respectively. The relationship of 0.5 > *y* > *z* implies that the fraction of vacancies is larger than this occurring due to the sole presence of the *Ln*O_1.5_ endmember. Thus, UO_1.5_ endmember must necessarily be present creating an additional fraction of vacancies. This also implies that in such a solid solution Ln occurs only as *Ln*O_1.5_. On the other hand, the relationship of *z* > *y* > 0 implies that the fraction of vacancies is smaller than this occurring due to the sole presence of *Ln*O_1.5_, thus, a certain fraction of *Ln*O_1.5_ is to be converted into *Ln*_0.5_U_0.5_O_2_. This second case is further split into the sub-cases IIa and IIb depending on *z* < 0.5 and *z* > 0.5. First, we consider the case I of 0.5 > *y* > *z.*

#### 2.1.3 Hypo-Stoichiometric Solid Solution, *z* < *y* < 0.5, Type I

The reference free energy is spanned by the three endmembers UO_2_, UO_1.5_, and *Ln*O_1.5_ according to the following equation

$$G_{\text{hypo},\text{I}}^{\text{ref}}=\sum_{i}X_{i}G_{i}$$ (18)

The excess free energy is given by

$$G_{\text{hypo},\text{I}}^{\text{exess}}=\sum_{j\neq i}X_{i}X_{j}\left(W_{ij}^{\text{h}}-TW_{ij}^{\text{s}}\right)$$ (19)

where $X_{\text{UO}2}=1-y\;,\;X_{\text{UO}1.5}=y-z,\;X_{Ln\text{O}1.5}=z$

The configurational entropy of this solid solution has two contributions. The already familiar contribution due to the mixing of vacancies and lattice O^2-^ anions is given by Eq. 10. The contribution due to the mixing of *Ln* ^+3^ with U^+4^, and U^+3^ requires a special consideration. In a solid solution, in which the radii of III- and IV-valent cations have similar sizes, the III-valent cations are expected to be associated with vacancies (Solomon et al., 2014). This association is modelled here by requesting the coordination number of III-valent cations to be 7. The consequence is that *Ln* ^+3^ can mix only with 7-fold U cations, the restriction that directly affects the configurational entropy. One can further note that the total fraction of 7-fold cations in such a solid solution is 2*y*. The structural formula becomes $\;{\text{U}}_{1-2y}^{4,8}{\text{U}}_{y}^{4,7}Ln_{z}^{3,7}{\text{U}}_{y-z}^{3,7}{\text{O}}_{2-0.5y}$ . This formula, in turn, implies that *Ln* ^+3^ can mix with U cations only over 2*y* sites. The configurational entropy is thus given by the following equation

$$S_{\text{hypo},\text{I}}^{\text{conf}}=-R\left(z\ln\left(\frac{z}{2y}\right)+\left(2y-z\right)\ln\left(\frac{2y-z}{2y}\right)\right)$$ (20)

A more detailed derivation of this equation is given in Supplementary Materials.

The total Gibbs free energy of the model is then given as

$$G_{\text{hypo},\text{I}}=G_{\text{hypo},\text{I}}^{\text{ref}}+G_{\text{hypo},\text{I}}^{\text{exess}}-T\left(S_{\text{OV}}^{\text{conf}}+S_{\text{hypo},\text{I}}^{\text{conf}}\right)$$ (21)

which is a function of *z*, *T*, $P_{{\text{O}}_{2}}$ , and *y*. The equilibrium relationship between all these parameters is obtained via the minimization of Eq. 21 with respect to *y*.

#### 2.1.4 Hypo-Stoichiometric Solid Solution, 0 < *y* < *z*, *z* < 0.5, Type IIa

In this case the endmember UO_1.5_ is absent, thus U^+5^ formation is allowed, and, thus, two *Ln*-bearing endmembers can co-exist. The reference and the excess free energies are given by the equations

$$G_{\text{hypo},\text{IIa}}^{\text{ref}}=\sum_{i}X_{i}G_{i}$$ (22)

and

$$G_{\text{hypo},\text{IIa}}^{\text{exess}}=\sum_{j\neq i}X_{i}X_{j}\left(W_{ij}^{\text{h}}-TW_{ij}^{\text{s}}\right)$$ (23)

where $X_{\text{UO}2}=1-y-2\left(z-y\right),\;X_{Ln0.5\text{U}0.5\text{O}2}=2\left(z-y\right),\;X_{Ln\text{O}1.5}=y$ .

As in the case I, we assume that III-valent cations, i.e. *Ln* ^+3^, are associated to vacancies. However, a part of *Ln* cations is involved in building the *Ln*_0.5_U_0.5_O_2_ endmember, which is intrinsically stoichiometric. Thus, it is natural to expect that a fraction *z*–*y* of *Ln* cations remains in 8-fold coordination. As the total fraction of 7-fold cations is 2*y*, a fraction of *y* U^+4^ cations need to be transferred into the 7-fold coordination. The structural formula takes the form $U_{1-2z}^{4,8}Ln_{z-y}^{3,8}U_{z-y}^{5,8}Ln_{y}^{3,7}U_{y}^{4,7}O_{2-0.5y}$ . The configurational entropy of cations has two contributions, one from cations in the 7-fold coordination and one from cations in the 8-fold coordination, which occur with the total fractions of 2*y* and 1–2*y*, respectively. Both contributions are combined below in one equation as follows

$$S_{\text{hypo},\text{IIa}}^{\text{conf}}=2Ry\ln2-R\left(\left(1-y-z\right)\ln\left(\frac{1-y-z)}{1-2y}\right)+\left(z-y\right)\ln\left(\frac{z-y}{1-2y}\right)\right)$$ (24)

The total Gibbs free energy of the model is then given as

$$G_{\text{hypo},\text{IIa}}=G_{\text{hypo},\text{IIa}}^{\text{ref}}+G_{\text{hypo},\text{IIa}}^{\text{exess}}-T\left(S_{\text{O}/\text{V}}^{\text{conf}}+S_{\text{hypo},\text{IIa}}^{\text{conf}}\right)$$ (25)

This model is valid for *z* < 0.5. When *z* = 0.5, all ${\text{U}}^{4,8}$ cations become exhausted. The next model overcomes this constraint by transferring a fraction of $Ln^{3,8}$ and ${\text{U}}^{5,8}$ cations into the 7-fold coordination.

#### 2.1.5 Hypo-Stoichiometric Solid Solution, 2*z* – 1 < *y* < 0.5, 0.5 < *z* < 0.75, Type IIb

When *z* > 0.5, the fraction of $Ln^{3,8}$ and the same fraction of ${\text{U}}^{5,8}$ can be written as *z* – *y* = (0.5–*y*) + (*z* – 0.5). If *z* > 0.5 this arrangement would violate the requirement that the total fraction of 8-fold cations is 1 – 2*y*. We assume that the fractions *z* – 0.5 of $Ln^{3,8}$ and *z* – 0.5 of ${\text{U}}^{5,8}$ cations are transferred to the 7-fold coordination. The structural formula takes the form $U_{1-2z+y}^{4,7}Ln_{y+z-0.5}^{3,7}U_{z-0.5}^{5,7}Ln_{0.5-y}^{3,8}U_{0.5-y}^{5,8}O_{2-0.5y}$ and the configurational entropy is given as:

$$S_{\text{hypo},\text{IIb}}^{\text{conf}}=-2R\left(0.5-y\right)\ln\frac{0.5-y}{1-2y}-R\left(\left(y+z-0.5\right)\ln\left(\frac{y+\;z-0.5}{2y}\right)+\left(y-z+0.5\right)\ln\left(\frac{y-z+0.5}{2y}\right)\right)$$ (26)

Other equations remain same as in the case IIa.

#### 2.1.6 Hyper-Stoichiometric Solution, Type III

Assuming that vacancies are absent at hyper-stoichiometric compositions, *Ln* ^+3^ can occur only as *Ln*_0.5_U_0.5_O_2_. Consequently, there are two fractions of U^+5^, a fraction of U^+5^, that is needed to balance *Ln* ^+3^, and a fraction of U^+5^ that is needed to balance O-interstitials. The reference Gibbs free energy and the excess Gibbs free energy are given as follows

$$G_{\text{hyper}}^{\text{ref}}=\sum_{i}X_{i}G_{i}$$ (27)

$$G_{\text{hyper}}^{\text{exess}}=\sum_{j\neq i}X_{i}X_{j}\left(W_{ij}^{\text{h}}-TW_{ij}^{\text{s}}\right)$$ (28)

where $X_{\text{UO}2}=1-2z-x,\;X_{\text{Ln}0.5\text{U}0.5\text{O}2}=2z,\;X_{\text{UO}2.5}=x$ .

Considering that *Ln*_0.5_U_0.5_O_2_ is intrinsically stoichiometric, all *Ln* ^+3^ cations and the equivalent fraction of U^+5^ cations are assumed to be in 8-fold coordination. The other U^+5^ cations that balance the interstitials may formally be prescribed coordination numbers larger than 8, because O-interstitials are expected to be in a close association to these cations due to the attraction of differently charged species. For simplicity we assign to all these atoms the 9-fold coordination. The structural formula of doped hyper-stoichiometric solid solution is $U_{1-x-2z}^{4,8}Ln_{z}^{3,8}U_{z}^{5,8}U_{x}^{5,9}O_{2+0.5x}$ .

As Ln^+3^ cations are assumed to preserve the coordination of 8, they can be mixed with U atoms only over the fraction 1 – *x* of 8-fold coordinated sites, thus the configurational entropy of cations is given by the equation

$$S_{\text{hyper}}^{\text{conf}}=-R\left(\left(1-x-z\right)\ln\left(\frac{1-x-z}{1-x}\right)+z\ln\left(\frac{z}{1-x}\right)\right)$$ (29)

The total Gibbs free energy of the model is then given as

$$G_{\text{hyper}}=G_{\text{hyper}}^{\text{ref}}+G_{\text{hyper}}^{\text{exess}}-T\left(S_{\text{Oi}/\text{Vi}}^{\text{conf}}+S_{\text{hyper}}^{\text{conf}}\right)$$ (30)

which is a function of *z*, *T*, $P_{{\text{O}}_{2}}$ , and *x*. An equilibrium relationship between all these parameters is obtained via the minimization of Eq. 30 with respect to *x*.

### 2.2 Structural Model

**FIGURE 2:**
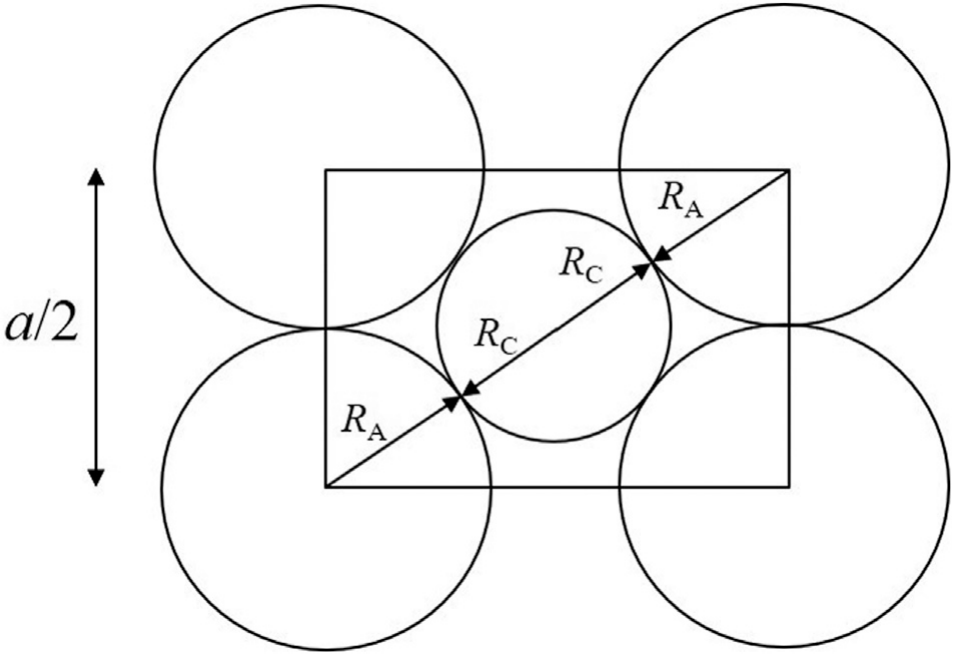
Geometric relationship used in the ion-packing model. The rectangle corresponds to the cross section of a cube composed of eight anions along its face diagonal. The spheres representing cations and anions are constrained to touch along the body diagonal. An additional condition (not used in the ion-packing model) could be set by requiring the spheres of anions to touch themselves along [100].

Previous research has shown that the lattice parameter of UO_2_ solid solutions varies as a function of the composition and non-stoichiometry, and that these variations could be predicted based on an ionic packing model (Ohmichi et al., 1981; Fukushima et al., 1983; Lee et al., 2016b; Bukaemskiy et al., 2021). This model utilizes a geometrical relationship between the lattice parameter, *a*, and the sum of the averaged radii of cations, $R_{\text{C}},$ and anions, $R_{\text{A}}$

$$a=\frac{4}{\sqrt{3}}\left(\langle R_{\text{C}}\rangle +\langle R_{\text{A}}\rangle \right),$$ (31)

which is determined by Figure 2.

The radii of cation/anions are further evaluated as sums all of cation/anion radii weighted by the cation/anion fractions that contribute to the structural formula, where the anions include the lattice oxygen O^2-^ and the oxygen vacancy

$$\langle R_{\text{A}}\rangle =\left(1-0.25y\right)R_{\text{O}}+0.25yR_{\text{V}}$$ (32)

$$\langle R_{\text{C}}\rangle =\sum_{C}\sum_{K}\sum_{i}X_{C}^{i,K}R_{C}^{i,K}$$ (33)

where $R_{C}^{i,K}$ and $X_{C}^{i,K}$ are the radius and the fraction of a *C* cation in *i* oxidation state and *K*-fold coordination and where $R_{\text{O}}$ and $R_{\text{V}}$ are the effective radii of the lattice oxygen anion and of the oxygen vacancy. The upper-case indices are used to characterize both the charge and the coordination number of a cation.

Following the study of Bukaemskiy et al. (Bukaemskiy et al., 2021) we assume that a vacancy has a defined radius that is larger than the ionic radius of oxygen. Such a model is consistent with the requirement that the average cation coordination number in hypo-stochiometric samples, K , is smaller than eight. An alternative description (Hong and Virkar, 1995; Marrocchelli et al., 2012; Marrocchelli et al., 2013) maintains the vacancy to be smaller than the radius of O^2-^, while keeping the coordination number of all cations equal to eight. We emphasize that in our approach the cation coordination numbers are made consistent not only with the condition of K  = 8 – 2*y*, but also with assumptions of short-range anion ordering. Interstitials do not contribute to Eq. 32. Thus, the average anion radius in the case of model III, where the vacancies are absent, is simply equal $R_{\text{O}}$ . The size effect of an interstitial is included into the effective radii of cations that occur in a close contact with the interstitial, i.e. into the effective radii of ${\text{U}}^{5,9}$ .

The radii of *Ln*, of O^2-^ and of a vacancy are adopted from our previous study on ZrO_2_—*Ln*O_1.5_ systems (Bukaemskiy et al., 2021). (Some radii in this set were slightly varied reflecting specific properties of UO_2_-based systems). The average radii can be trivially computed from the structural formulas corresponding to the models introduced above provided that the parameters *x*, *y*, and *z* are known. The relevant structural formulas and the equations to calculate average cation radii are given in Table 1.

**TABLE 1:** Structural formulas of the solid solution models and the expressions to calculate average cation radii.

	Structural formula	$R_{C}$
I	$U_{1-2y}^{4,8}U_{y}^{4,7}Ln_{z}^{3,7}U_{y-z}^{3,7}O_{2-0.5y}$	$\left(1-2y\right)R_{\text{U}}^{4,8}+yR_{\text{U}}^{3,7}+zR_{Ln}^{3,7}+\left(y-z\right)R_{\text{U}}^{3,7}$
IIa	$U_{1-2z}^{4,8}Ln_{z-y}^{3,8}U_{z-y}^{5,8}Ln_{y}^{3,7}U_{y}^{4,7}O_{2-0.5y}$	$\left(1-2z\right)R_{\text{U}}^{4,8}+y\left(R_{\text{U}}^{4,7}+R_{Ln}^{3,7}\right)+\left(z-y\right)\left(R_{\text{U}}^{3,8}+R_{\text{U}}^{5,8}\right)$
IIb	$U_{1-2z+y}^{4,7}Ln_{y+z-0.5}^{3,7}U_{z-0.5}^{5,7}Ln_{0.5-y}^{3,8}U_{0.5-y}^{5,8}O_{2-0.5y}$	$\left(1-2z+y\right)R_{\text{U}}^{4,7}+\left(y+z-0.5\right)R_{Ln}^{3,7}+\left(z-0.5\right)R_{\text{U}}^{5,7}+\left(0.5-y\right)\left(R_{Ln}^{3,8}+R_{\text{U}}^{5,8}\right)$
III	$U_{1-2z-x}^{4,8}Ln_{z}^{3,8}U_{z}^{5,8}U_{x}^{5,9}O_{2+0.5x}$	$\left(1-2z-x\right)R_{\text{U}}^{4,8}+z\left(R_{Ln}^{3,8}+R_{\text{U}}^{5,8}\right)+xR_{\text{U}}^{5,9}$

The thermodynamic model predicts the equilibrium *x* and *y* parameters as functions of *z*, *T,* and $P_{{\text{O}}_{2}}$ . Consequently, the lattice parameter is also a function of *z*, *T,* and $P_{{\text{O}}_{2}}$ . Conveniently, for the relevant systems of *Ln* = {La, Pr, Nd, Gd} the data cover a range of synthesis conditions, while it is usually possible to distinguish limiting states with well-defined stoichiometric relations, such as the strictly stoichiometric case (*y* = *x* = 0, the fractions of *Ln* ^+3^ and U^+5^ are equal) and the fully reduced hypo-stoichiometric case (U^+5^ is absent, *y* = *z*). The available data were used to fit either the radius of U^+5^ or refine the radius of a vacancy. The cation radii of a fraction of U atoms in hyper-stoichiometric systems are affected by an unknown effect of addition of O-interstitials. Here we introduced a hypothetic U^+5^ cation in 9-fold coordination to reflect the effect of these extra oxygen atoms on the lattice parameter. The radius of ${\text{U}}^{5,9}$ has been determined via a fit to available data on the dependence of the lattice parameter of pure UO_2+0.5*x*_ on *x* for *x* < 0.30.

## 3 Results and Discussion

### 3.1 Thermodynamic Model

**FIGURE 3:**
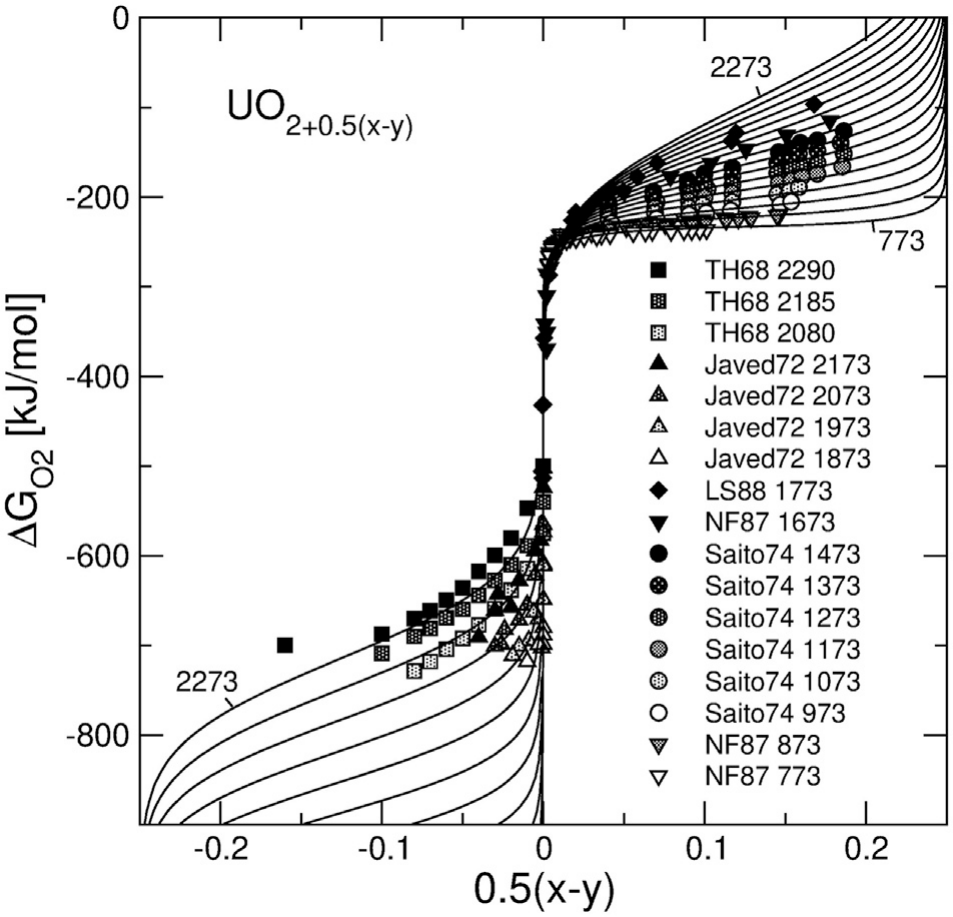
Oxygen chemical potential plotted vs. non-stoichiometry parameter of UO_2+0.5(*x*-*y*)_. Solid lines are the isotherms predicted with the model. The temperature range is from 773 to 2273 K. The experimental data are from Tetenbaum and Hunt (Tetenbaum and Hunt, 1968), Javed (Javed, 1972), Lindemer and Sutton (Lindemer and Sutton, 1988), Nakamura and Fujino (Nakamura and Fujino, 1987) and Saito (Saito, 1974).

Figure 3 shows the results of the model fit to $\Delta G_{{\text{O}}_{2}}=RT\log\left(P_{{\text{O}}_{2}}/P^{0}\right)$ vs. non-stoichiometry data for pure UO_2+0.5(*x-y*)_. Importantly, good fits to both hypo- and hyper-stoichiometric samples were achieved with a minimum set of adjustable parameters. An important observation is that for a given temperature in hyper-stoichiometric domain the oxygen chemical potential increases at high degrees of non-stoichiometry. Thus, the higher the *x* value, the more stable is UO_2_ against a further oxidation. This effect is reflected in the positive values of $W_{23}^{\text{h}}$ and $W_{23}^{\text{s}}\;$ parameters that describe the interaction between UO_2_ and UO_2.5_. The values of all fitted parameters are given in Tables 2, 3, 4. Although our model does not explicitly include disproportionation (Eqs. 12, 13), the parameters in Table 2 allow an estimation of their combined effect. The difference between $\Delta G_{\text{UO}1.5}^{0}$ and $\Delta G_{\text{UO}2.5}^{0}$ of 412 kJ/mol (∼4.3 eV) is marginally consistent with the value of ∼3.5 eV that is composed of ab initio computed defect formation energies (Nerikar et al., 2009; Cooper et al., 2018). The values in Table 2 show also that an insertion of oxygen interstitials coupled with an oxidation of two U^+4^ cations into U^+5^ cations (the formation reaction of UO_2.5_), as well as a creation of an oxygen vacancy coupled with a reduction of two U^+4^ cations into U^+3^ cations (the formation of UO_1.5_), are associated with positive entropy effects. The formation of the *Ln*_0.5_U_0.5_O_2_ endmember from UO_2_ and *Ln*O_1.5_ is also associated with a positive entropy effect. The standard Gibbs free energy of the UO_2.5_ endmember is predicted to be more negative than this of UO_2_ consistently with the instability of UO_2_ in air. The Gibbs free energies of *Ln*_0.5_U_0.5_O_2_ endmembers are assessed to be ∼60 kJ/mol more negative than the half sum of the free energies of UO_2_ and *Ln*O_1.5_, consistently with the observation that the formation of hypo-stoichiometric samples along with the vacancy forming mechanism (Eq. 14) becomes possible only at a rather low oxygen pressure.

**TABLE 2:** Fitted standard state parameters defining the thermodynamic properties of the endmembers.

	*i*	$\Delta G_{i}^{0}$ (kJ/mol)	$\Delta S_{i}^{0}$ (J/K/mol)
UO_2_	2	0	0
*Ln*O_1.5_	4	0	0
UO_1.5_	1	336.9	16.7
UO_2.5_	3	−75.0	30.6
Gd_0.5_U_0.5_O_2_	5	−64.65	6.0
Nd_0.5_U_0.5_O_2_	5	−63.3	12.0
Pr_0.5_U_0.5_O_2_	5	−60.4	16.0
La_0.5_U_0.5_O_2_	5	−56.25	10.0

**TABLE 3:** Margules interaction parameters in kJ/mol determined via a model fit to the thermodynamic data.

	*i*	*j*	$W_{ij}^{\text{h}}\;$	$W_{ij}^{\text{s}}\;$
UO_2_—UO_2.5_	2	3	24.37	18.75
UO_2_—UO_1.5_	2	1	17.30	11.9
Gd_0.5_U_0.5_O_2_—UO_2.5_	5	3	40.0	0
Nd_0.5_U_0.5_O_2_—UO_2.5_	5	3	35.0	0
Pr_0.5_U_0.5_O_2_—UO_2.5_	5	3	30.0	0
La_0.5_U_0.5_O_2_—UO_2.5_	5	3	15.0	0
GdO_1.5_—UO_2_	4	2	60.0	0
NdO_1.5_—UO_2_	4	2	40.0	0
PrO_1.5_—UO_2_	4	2	60.0	0
LaO_1.5_—UO_2_	4	2	70.0	0
*Ln*_0.5_U_0.5_O_2_—UO_2_	5	2	0	0
*Ln*O_1.5_—UO_1.5_	4	1	0	0
*Ln*_0.5_U_0.5_O_2_—*Ln*O_1.5_	5	4	0	0

**TABLE 4:** Cation radii (in Å) accepted in the study.

$C^{i}$	$R_{C}^{i,7}$	$R_{C}^{i,8}$	$R_{C}^{i,9}$
Gd^3^	1.0012	1.0537	
Nd^3^	1.0476	1.0994	
Pr^3^	1.0680	1.1190	
La^3^	1.1014	1.1665	
U^3^	1.0995	1.1547	
U^4^	0.9400	0.9952	
U^5^	0.8400	0.8635	0.9760

**FIGURE 4:**
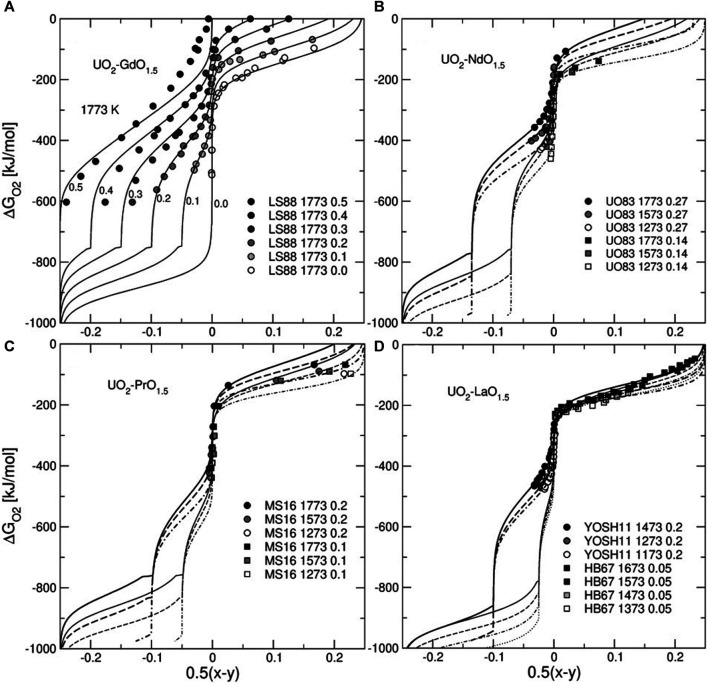
Oxygen chemical potential plotted vs. non-stoichiometry parameter of U_1-*z*_ *Ln*_*z*_O_2+0.5(*x-y*)_. Lines are the isotherms predicted with the model. The temperature ranges in (b), (c), and (d) span the synthesis temperature interval given in the legend (the solid and the dotted lines correspond to the highest and lowest temperatures, respectively). The experimental data are from Lindemer and Sutton (Lindemer and Sutton, 1988), Une and Oguma (Une and Oguma, 1983a), McMurray and Silva (McMurray and Silva, 2016), Yoshida et al. (Yoshida et al., 2011) and Hagemark and Broli (Hagemark and Broli, 1967). Note an increase in the magnitude of the composition effect on the chemical potential of O_2_ in the direction from La to Gd.

Figure 4 shows ∆ $G_{{\text{O}}_{2}}$ vs. non-stoichiometry plots for the Ln-doped systems. The model predicts different modes of the dependence of ∆ $G_{{\text{O}}_{2}}$ on non-stoichiometry. The transitions between the modes occur at *y* = *z* and at *y* = *x* = 0. These boundaries correspond to states that are common to models I and II and II and III, respectively. The lowest branch of the ∆ $G_{{\text{O}}_{2}}$ curve corresponds to the oxidation of U^+3^ to U^+4^. An important property of UO_2_—*Ln*O_1.5_ systems is a two-step oxidation of U^+4^ to U^+5^. The first oxidation step starts at *y* = *z* and ends at *y* = 0. At this step the formation of U^+5^ is balanced by extra oxygen anions that fill available oxygen vacancies. Thermodynamically, this process is reflected in the gradual increase in the fraction of the stoichiometric endmember *Ln*_0.5_U_0.5_O_2_. This process ends after all vacancies are filled in and the solution becomes stoichiometric. The second oxidation step starts at a much higher oxidation potential within a stoichiometric solid solution. The formation of U^+5^ is then balanced by oxygen anions that fill interstitial sites. This second oxidation step requires a higher oxidation potential in samples containing more *Ln*_0.5_U_0.5_O_2_. Thus, the *Ln*-doping in this region stabilizes the solid solution thermodynamically against the oxidation. This effect is particularly strong in the Gd—U system and is very weak in the system of La—U. The different behaviour correlates with the Margules interaction parameter for the *Ln*_0.5_U_0.5_O_2_—UO_2.5_ binary. The value of this parameter decreases strongly in the row of Gd > Nd > Pr > La. A possible reason for this effect is discussed further in the text.

### 3.2 Structural Model

**FIGURE 5:**
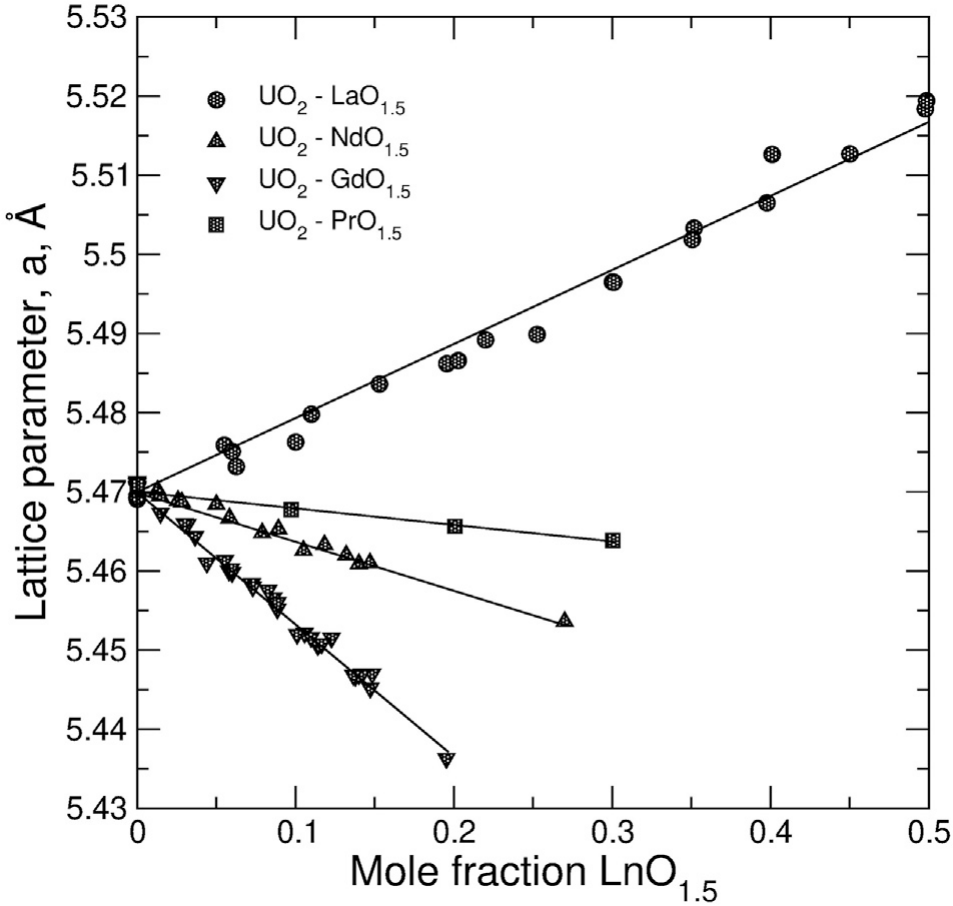
The dependence of the lattice parameter in stoichiometric UO_2_–*Ln*O_1.5_ systems on the composition. The data are fitted to linear equations to determine the radii of ${\text{U}}^{4,8}$ , $Ln^{3,8}$ , and ${\text{U}}^{5,8}$ ,. The experimental data are from (La) Hill (Hill, 1962), Stadlbauer et al. (Stadlbauer et al., 1974), Preiur et al. (Prieur et al., 2018); (Pr) Yamashita et al. (Yamashita et al., 1985); (Nd) Une and Oguma (Une and Oguma, 1983a), Ohmichi et al. (Ohmichi et al., 1981), Fukushima et al. (Fukushima et al., 1983); (Gd) Baena et al. (Baena et al., 2015), Ohmichi et al. (Ohmichi et al., 1981), Cardinaels et al. (Cardinaels et al., 2012), Leyva et al. (Leyva et al., 2002), Fukushima et al. (Fukushima et al., 1982), Hirai and Ishimoto (Hirai and Ishimoto, 1991), Soldati et al. (Soldati et al., 2016).

Figure 5 displays the dependence of the lattice parameter in stoichiometric UO_2_—*Ln*O_1.5_ systems. Linear relationships were assumed to determine (refine) values of cation radii in 8-fold coordination. Most of the values remain close to Shannon’s ionic radii (Shannon, 1976). The size of the oxygen anion of 1.3736 Å was adopted from Bukaemskiy et al. (Bukaemskiy et al., 2021) as fitted to data on ZrO_2_-based systems. The radii of cations in the 7-fold coordination are also from (Bukaemskiy et al., 2021). The effective size of the vacancy of 1.54 Å is slightly increased relative to the value of 1.53 ± 0.02 (Bukaemskiy et al., 2021) to give a better description of data on hypo-stoichiometric UO_2_-based solids solutions (Hill, 1962; Wadier, 1973), which are discussed below.

The hyper-stoichiometric domain (0 < *x* < 0.3) is successfully described with a model in which U^+5^ cations that are needed for charge balancing the interstitials are assumed to be 9-fold coordinated with the ionic radius of 0.976 Å. This is reasonable assumption considering the lack of data. The interstitial oxygen is assumed to have no direct influence on the lattice parameter. Indeed, the size of an interstitial cannot be simply reflected within the ion-packing concept, because its structural position does not comply with a regular position of a cation or an anion. Thus, its effect is mapped onto the effective radius of ${\text{U}}^{5,9}$ . The radii of ${\text{U}}^{3,7}$ and of ${\text{U}}^{3,8}$ were assumed to vary with the same slope vs. the coordination number as the radii of ${\text{U}}^{4,7}$ and of ${\text{U}}^{4,8}$ . The absolute values were increased to fit two data points from Anderson (Anderson et al., 1960) (not shown here). We note that the radii of ${\text{U}}^{3,7}$ and of ${\text{U}}^{3,8}$ adopted here have very large uncertainty, as there are almost no experimental data to be used as constraints. The set of the cation radii is given in Table 4.

**FIGURE 6:**
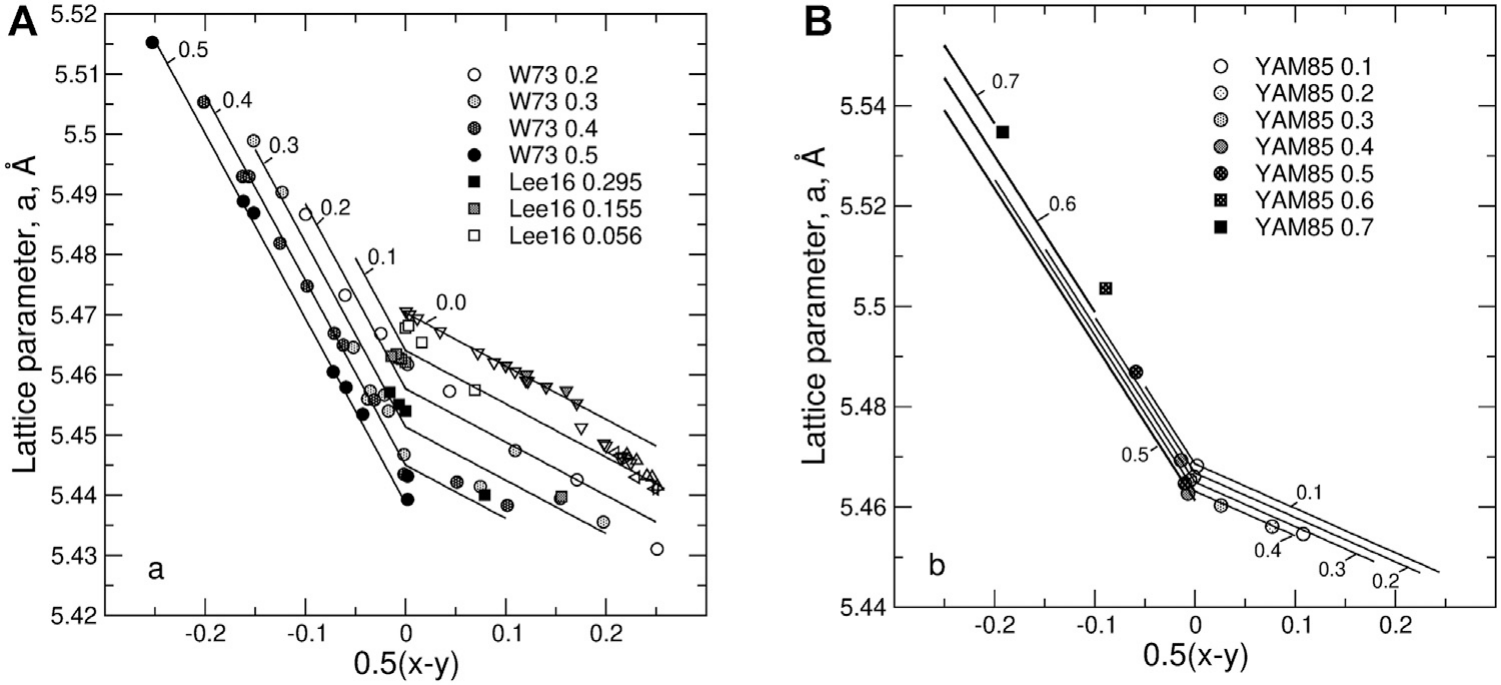
The dependence of the lattice parameter on non-stoichiometry, 0.5(*x-y*), and *z* in **(A)** U_1-*z*_Nd_*z*_O_2+0.5(*x*-*y*)_ and **(B)** U_1-*z*_Pr_*z*_O_2+0.5(*x*-*y*)_ systems. Solid lines are compositional isopleths predicted with the model. The experimental data (circles and squares) are from Wadier (Wadier, 1973) (circles), Lee et al. (Lee et al., 2016b) (squares), Yamashita et al. (Yamashita et al., 1985) (circles and squares are the samples synthesized at 1623 K), Lynds et al. (Lynds et al., 1963) (open down triangles), Belbeoch (Belbeoch et al., 1964) (shaded down triangles), Matsui and Naito (Matsui and Naito, 1975) (open up triangles), Schaner (Schaner, 1960) (left triangles), Grønvold (Grønvold, 1955) (shaded up triangles). The data on pure UO_2_ obviously fall on two trends. The first trend that extends from the stoichiometric UO_2_ up to *x* ∼ 0.30 is assumed to represent the fluorite-type solid solution. The trend extending to larger *x* values is assumed to represent U_4_O_9_-type structure. Thus, only the data from the first trend were used in the fit.

Figure 6 shows the predicted dependence of the lattice parameter in UO_2_—PrO_1.5_ and UO_2_—NdO_1.5_ on the composition and on non-stoichiometry. An increase in the hyper-stoichiometry causes a linear decrease of the lattice parameter. Clearly, the composition dependence of the lattice parameter is significantly less pronounced in the case of Pr—U system than in the case of Nd—U system. This is because the half sum of the radii of 8-fold Pr^+3^ and U^+5^, is almost equal to the radius of U^+4^ cation.

### 3.3 Structural-Thermodynamic Model

**FIGURE 7:**
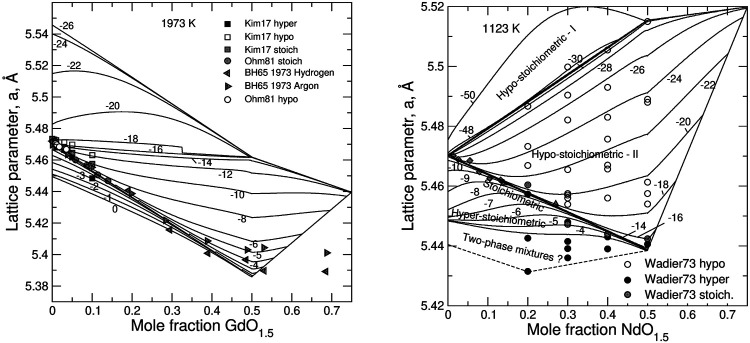
Relationship between the lattice parameter and the degree of doping, *z*, in GdO_1.5_—UO_2_ **(left)** and NdO_1.5_–UO_2_ **(right)** systems. Solid lines are calculated with the model isopleths of constant $\log\left(P_{{\text{O}}_{2}}/P^{0}\right)$ . Dashed lines schematically outline the two-phase field, where fluorite solid solution likely coexists with U_4_O_9_-type solid solution. The fluorite phase is subdivided into three fields which correspond to hypostoichiometric I, hypo-stoichiometric II and hyper-stoichiometric solid solutions for which different models are implemented. The hypo-stoichiometric type-II field is further subdivided into II,a and II,b areas by the line *z* = 0.5. The experimental data are from Kim et al. (Kim et al., 2017), Ohmichi et al. (Ohmichi et al., 1981), Beals and Handwerk (Beals and Handwerk, 1965), Wadier (Wadier, 1973). The data falling outside the quadrilateral possibly indicate the need of including of U^+6^ states into the model and/or the need of taking into account the presence of an additional phase.

**FIGURE 8:**
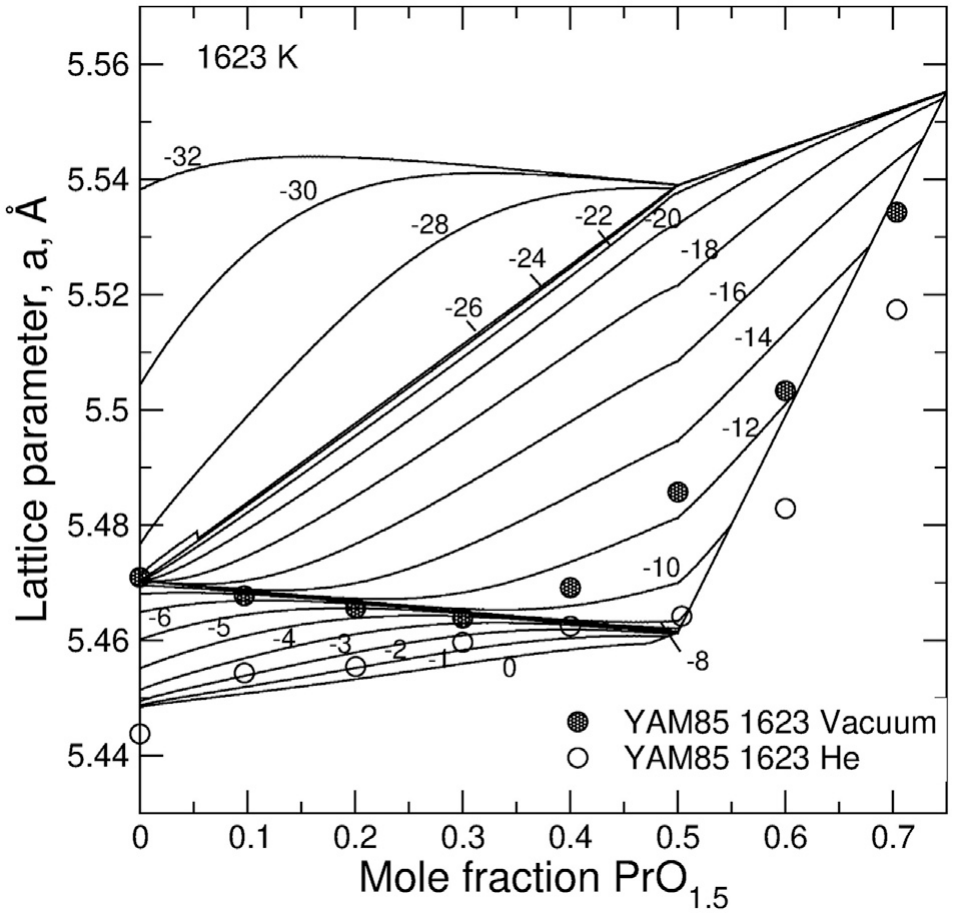
Relationship between the lattice parameter and the degree of doping, *z*, and $\log\left(P_{{\text{O}}_{2}}/P^{0}\right)$ in PrO_1.5_-UO_2_ system predicted from the model. The experimental data series from Yamashita et al. (Yamashita et al., 1985) are interpreted to be synthesized at $\log\left(P_{{\text{O}}_{2}}/P^{0}\right)$ of –2 and –12.

**FIGURE 9:**
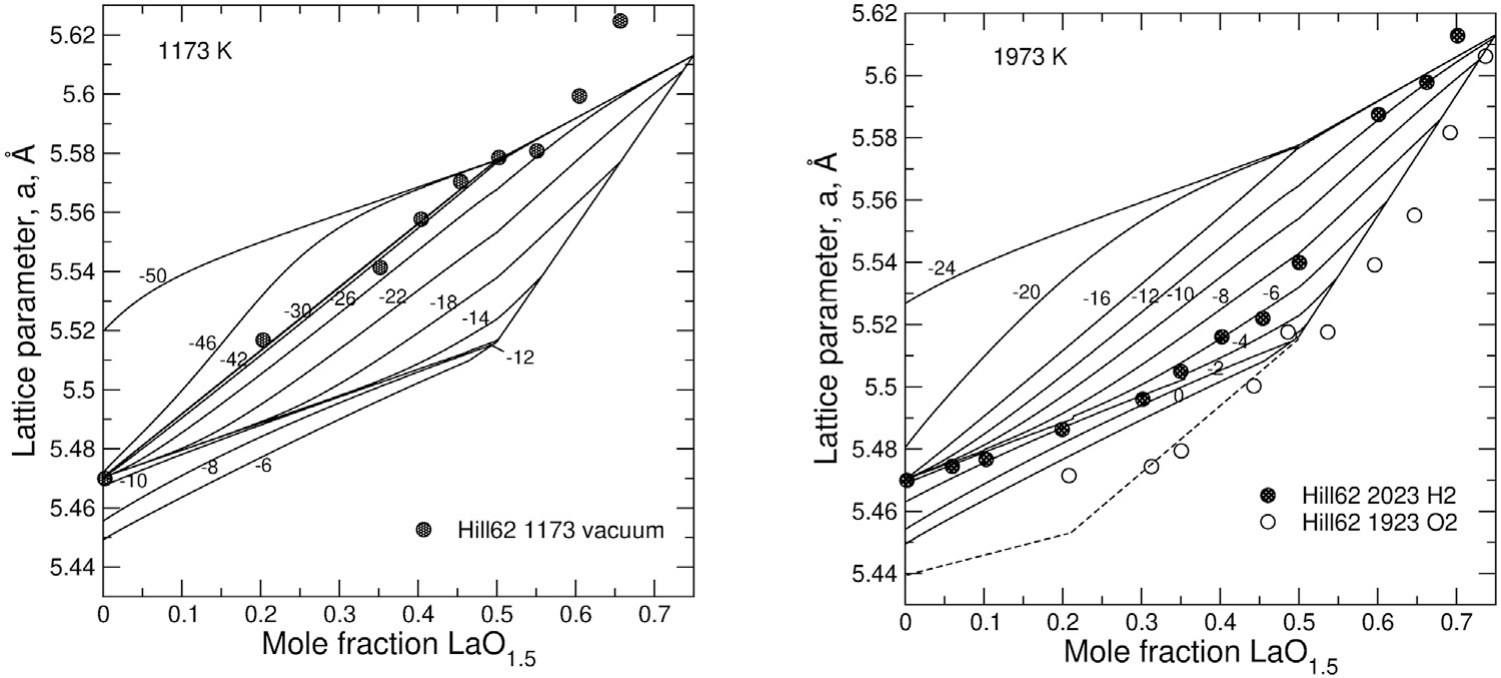
Relationship between the lattice parameter and the degree of doping, *z*, in LaO_1.5_–UO_2_ system predicted from the model. The experimental data series from Hill (Hill, 1962) are interpreted to be synthesized at –42 < $\log\left(P_{{\text{O}}_{2}}/P^{0}\right)$ < –26 **(left)** and at $\log\left(P_{{\text{O}}_{2}}/P^{0}\right)$ of –6 and 0 **(right)**. The data falling outside the quadrilateral possibly indicate the need of including U^+6^ states into the model.

The thermodynamic relationships allow the prediction of *x* and *y*, as functions of *z*, *T,* and $P_{{\text{O}}_{2}}$ . Consequently, the lattice parameter is also a function of *z*, *T* and $P_{{\text{O}}_{2}}$ . This circumstance allows the computation of $\log\left(P_{{\text{O}}_{2}}/P^{0}\right)$ isopleths as functions of *a* and *z*. Examples of such calculations are given in Figures 7–9. These figures can be used to reconstruct synthesis conditions from the data on the lattice parameter variation with *z*. While an experimental description could be limited by indicating only a type of a gas used in the synthesis, the present diagrams allow a more precise characterisation of the synthesis conditions in terms of the oxygen partial pressure. The knowledge of a detailed relationship between *a*, *x*, *y*, *z*, *T,* and $P_{{\text{O}}_{2}}$ appears to be very useful in designing experiments allowing synthesis of samples with required type and degree of non-stoichiometry. By measuring the lattice parameter and by comparing it with the predicted value, there appears a possibility of controlling the thermodynamic equilibration in synthesis experiments. This is particularly important in experiments aiming in distinguishing between various factors affecting the resistance of *Ln*-doped UO_2_ to oxidation and to oxidative dissolution. The computed diagrams are particularly instructive for predicting conditions that allow the synthesis of stoichiometric samples. For example, from Figure 7 one can deduce that the synthesis of stoichiometric samples in NdO_1.5_—UO_2_ system can be performed at *T* = 1123 K and –14 < $\log\left(P_{{\text{O}}_{2}}/P^{0}\right)$ < –12, while the developed computer program allows predicting the necessary oxygen partial pressures at any other temperature. Importantly, the slope, d*a*/d*z*, within the row of stoichiometric samples depends on the type of Ln (Figure 5). Zero slope is determined by the equation $R_{Ln}^{3,8}$ + $R_{\text{U}}^{5,8}$ = 2 $R_{\text{U}}^{4,8}$ which is nearly exactly fulfilled for Pr. UO_2_—LaO_1.5_ is the only system characterized by a positive slope. Remarkably, on the thermodynamic level, the UO_2_—LaO_1.5_ system is characterized by the lowest increase in $\log\left(P_{{\text{O}}_{2}}/P^{0}\right)$ with the doping level (Figure 4D), which correlates with the low value of the Margules interaction between La_0.5_U_0.5_O_2_ and UO_2.5_ endmembers (Table 2). Recalling that a Margules parameter measures the size mismatch between two endmembers (Vinograd et al., 2013; Kowalski and Li, 2016), the low value of $W^{\text{h}}$ in the case of La_0.5_U_0.5_O_2_—UO_2.5_ is counterintuitive. Indeed, in the case of La_0.5_U_0.5_O_2_ the size mismatch between *Ln*_0.5_U_0.5_O_2_ and UO_2.5_ is maximized. The size mismatch between these endmembers occurs primarily due to the difference in the radii of ${\text{La}}^{3,8}$ and ${\text{U}}^{5,9}$ . The local strain is caused due to a random formation of ${\text{La}}^{3,8}$ - ${\text{La}}^{3,8}$ and ${\text{U}}^{5,9}$ - ${\text{U}}^{5,9}$ , pairs, which are either too large or too small to fit the common average distance along [111] (Figure 2). The $W^{h}\;$ parameter of *Ln*_0.5_U_0.5_O_2_—UO_2.5_ interaction reflects this local strain effect due to the misfit between ${\text{La}}^{3,8}$ and ${\text{U}}^{5,9}$ . Likewise, the $W^{h}$ parameter of UO_2_—UO_2.5_ interaction reflects the misfit between ${\text{U}}^{4,8}$ and ${\text{U}}^{5,9}$ . The counterintuitive variation of the Margules *Ln*_0.5_U_0.5_O_2_—UO_2.5_ interactions can be qualitatively explained under an assumption that these parameters, besides the local strain along [111], reflect an additional strain effect, that is caused by an overlap of anion spheres along [100]. Indeed, in the case of pure stoichiometric UO_2_ the condition of a close touch between anion spheres along [100] corresponds to the lattice parameter of 5.494 Å (Figure 2). The lattice parameter of ∼5.47 Å of stoichiometric UO_2_ already corresponds to a slight overlap of the anion spheres. Obviously, the lattice contraction to values significantly smaller than ∼5.47 Å would cause an increasing strain along [100], as the ionic spheres would be overlapped/compressed more significantly. A normal structural reaction to such an overlap is the tendency of a cation to decrease its coordination number, as happens, for example, in monoclinic ZrO_2_. Thus, the overlap is a sign of a decreased stability of the fluorite phase. Importantly, the overlap of anions is a global effect, to which all endmembers contribute, while their contributions could be of different magnitude and sign. Effectively, an addition of La_0.5_U_0.5_O_2_ decreases the overlap of anion spheres (causing a negative contribution to the strain energy), while an addition of Nd_0.5_U_0.5_O_2_ and Gd_0.5_U_0.5_O_2_ makes the overlap stronger. The overlap also increases due to an increase in the fraction of UO_2.5_ (i.e. due to the oxidation). Assuming the Margules parameters reflect both [111] and [100] strains, the low value of the La_0.5_U_0.5_O_2_—UO_2.5_ interaction parameter can be easily rationalized as a superposition of two effects of different signs cancelling each other to a large extent. Considering this hypothesis, an addition of La should make the oxidation of UO_2_ easier, as it decreases the overlap allowing for the lattice contraction, while an addition of Gd or Nd should make it more difficult.

Our hypothesis is that the stabilizing effect of *Ln*-doping in the case of Nd, Gd (and smaller *Ln*) is primarily caused by the lattice contraction due to the doping and by the increasing strain energy cost of an additional contraction (additional overlap of oxygen anions along [100]) required by the oxidation. At the same time, an increase in the oxygen chemical potential with the extent of hyper-stoichiometry observed in both undoped and doped systems (Figures 3, 4) can be equally well rationalized. As the oxidation from UO_2_ to UO_2+0.5*x*_ (*x* < 0.5) causes an unfavourable decrease in the lattice parameter, the oxidation (i.e. an increase in *x*) is a self-inhibiting process. This self-inhibition effect could be responsible for the decrease in the U^+6^ yields after repeating exposures to H_2_O_2_ observed recently in oxidative dissolution experiments (Maier et al., 2020).

Further development of the model would require extending it to *Ln*-dopants with the cation radii smaller than this of Gd^+3^, to Y and to di-valent dopants. An explicit introducing of U_1/3_ *Ln*_2/3_O_2_ and UO_3_ endmembers would probably cure apparent problems of the present model at *z* > 0.5.

## 4 Conclusion

The data on the dependence of the oxygen potential of doped UO_2_ solid solutions on the composition, temperature and non-stoichiometry are linked to the data on the dependence of the lattice parameter on composition and non-stoichiometry within the same model frame. This brings up a possibility of relating changes in the lattice parameter to *T* and $\log\left(P_{{\text{O}}_{2}}/P^{0}\right)$ . As the lattice parameter can be predicted as a function of synthesis conditions, its measuring provides a test for an attainment of the thermodynamic equilibrium in a particular experiment.

Good fit of the model to available structural data on the dependence of the lattice parameter, *a*, on *z*, *x,* and *y* shows that the model assumptions of short-range ordering, such as the vacancy-vacancy avoidance, the avoidance of di-interstitial clusters, the association of vacancies to III-valent cations and the association of U^+5^ to interstitial O^2-^ are reasonable. These assumptions have an important effect on the structural model by limiting the spectrum of cation coordination numbers and thus allowing for the application of the ion-packing concept.

The SRO also imposes a profound effect on the thermodynamic model causing a significant reduction in the configurational entropy relative to the ideal mixing model. This model feature is essential for achieving a good fit to $\log\left(P_{{\text{O}}_{2}}/P^{0}\right)$ vs. non-stoichiometry data.

The entropy decreases 1) due to the ordering of vacancies and interstitials, 2) due to the cation-anion association effects and 3) due to the neglect of the configurational effect from U^+4^/U^+3^ and U^+4^/U^+5^ mixing. The achieved consistency between the structural and thermodynamic descriptions provides an argument in favour of the validity of these assumptions. Further experimental and computational studies aiming at testing these assumptions in other similar systems would be desirable.

The developed models are thermodynamically simple and transparent. An introduction of a new chemical component, such as *Ln*O_1.5_, requires just two parameters to define the standard thermodynamic properties of a *Ln*_0.5_U_0.5_O_2_ endmember and two Margules parameters to model the interactions *Ln*_0.5_U_0.5_O_2_—UO_2.5_ and *Ln*O_1.5_—UO_2_. This offers a great advantage over models based on the Compound Energy Formalism (Saunders and Miodownik, 1998; Hillert, 2001) that require a larger number of adjustable parameters. The present model requires the Gibbs free energy minimization with respect to just one parameter, *y* or *x*, in each model domain.

The small number of thermodynamic parameters provides a possibility of identifying main factors that are responsible for the dependence of the oxidation potential on the doping and on the extent of oxidation. In hyper-stoichiometric region these are the Margules parameters for the interactions between *Ln*_0.5_U_0.5_O_2_ and UO_2.5_ and between UO_2_ and UO_2.5_. As the first parameter correlates with d*a*/d*z* slope, the strain associated with the lattice contraction appears to be the likely cause of the stabilization of doped samples against the oxidation.

The proposed mechanism of the resistance of *Ln*-doped UO_2_ to the thermodynamically controlled oxidation may be equally applicable to more complex UO_2_-based simulated spent fuel, as it is known that the lattice parameter of simulated fuel (e.g. UO_2_ + Zr, Ce, Pr, Nd, and Y) decreases with the simulated burn-up (Une and Oguma, 1983b).

The thermodynamic and structural models developed here provide a possible explanation for the observed corrosion resistance of *Ln* doped UO_2_ compared to pure UO_2_ under conditions expected in a deep geological repository for spent nuclear fuel. However, with respect to the corrosion of SNF under disposal conditions other effects (e.g., radiation effects, He build up, effects of other non-*Ln* fission products) need to be taken into account.

## Data Availability

The original contributions presented in the study are included in the article/Supplementary Material, further inquiries can be directed to the corresponding author.
