# Supplementary material for: Thermodynamic and Structural Modelling of Non-Stoichiometric Ln-Doped UO2 Solid Solutions, Ln = {La, Pr, Nd, Gd}
Source: Front Chem. 2021 Nov 8;9:705024. doi: 10.3389/fchem.2021.705024 (PMC8637892; doi:10.3389/fchem.2021.705024)
Supplement: Supplementary file 1 [file DataSheet1.docx]

**Thermodynamic and structural modelling of non-stoichiometric *Ln*-doped UO_2_ solid solutions*, Ln* = {La, Pr, Nd, Gd}**

**Supplementary Materials**

**V.L. Vinograd*, A.A. Bukaemskiy, G. Modolo, G. Deissmann and D. Bosbach**

Forschungszentrum Jülich, Nuclear Waste Management & Reactor Safety, Institute of Energy & Climate Research IEK 6

*** Correspondence:**V.L. Vinograd
v.vinograd@fz-juelich.de

1. **Derivation of expressions for the configurational entropy for UO_2+0.5(_*_x-y_*_)_**

The simplest model for the configurational entropy assumes ideal mixing of all cation species on the cation site, the ideal mixing of vacancies and lattice oxygen atoms on the anion site and the ideal mixing of interstitials with vacant interstitials on the interstitial site. This model does not fit the oxygen potential data leading to a too strong stabilization of non-stoichiometric compositions. Thus, certain short-range order restrictions leading to a reduction in the configurational entropy should be guessed. The first guess is that vacancies, due to their effective positive charge, repel each other at short distances thus limiting the effective fraction of anion sites over which vacancies (V) and lattice oxygen atoms (O) can mix. Our previous studies on ZrO_2_-*Ln*O_1.5_ systems [1,2] at low fractions of *Ln*O_1.5_ favoured a model in which vacancies repel each other up to the third nearest-neighbour distance, such that vacancy-vacancy pairs are allowed to be formed only at the fourth-neighbour and larger distances. Such a V/O distribution can be approximately modelled under the assumption that vacancies occur (and mix with lattice oxygen) within an imaginary sublattice in which the nearest-neighbour distance corresponds to the fourth-nearest distance of the original anion lattice. Such a sublattice can be visualized as simple cubic lattice with a lattice vector ***a**=2*a*** that is twice the lattice vector ***a*** = *a*/2 of the original anion lattice, where *a* is the parameter of the fluorite cell. Such a sublattice contains 1/8 of the original anion sites. As the concentration of vacancies in the original lattice is 0.25*y*, the vacancy concentration within the simple cubic sublattice is 2*y*, i.e. eight times larger. Noting that the total number of anion sites is 2, the fraction of anion sites over which the mixing can occur is eight times smaller, i.e. 0.25. Thus, the expression for the configurational entropy (per one mole of cations or per two mole of anions) is

$S_{O/V}^{\mathrm{conf}}=-0.25R(2y\ln\left( 2y \right)+\left( 1-2y \right)\ln(1-2y))$. (1)

This equation is valid for 0 < *y* < 0.5. Thus, we limit the model applicability only to cases where the concentration of vacancies does not exceed 1/8 of the total anion sites.

Noting the symmetry in the shape of the dependence of the oxygen potential in UO_2+0.5(_*_x-y_*_)_ on *y* and on *x*, we made a further assumption that the mixing of interstitials (O_i_) with vacant interstitials (V_i_) is given by an analogous equation

$S_{Oi/Vi}^{\mathrm{conf}}=-0.25R(2x\ln\left( 2x \right)+\left( 1-2x \right)\ln(1-2x))$, (2)

and using (1) and (2) got a very good fit to all available oxygen potential data on UO_2+0.5(_*_x-y_*_)_. The equation (2) can be explained under the assumption that oxygen interstitials are allowed to make pairs, while these pairs repel each other, such that there are no nearest-neighbour contacts between the pairs. These isolated pairs can be arranged over the sublattice of interstitials forming another sublattice that covers ¼ of all interstitial sites. The model composed of pairs of interstitials agrees with some recent structure modelling studies discussed in the main text. As the total fraction of interstitials is 0.5*x*, the fraction of interstitials within the sublattice is 2*x*. Since the total number of interstitials per 2 lattice oxygen sites is 1, the fraction of sites over which the mixing of interstitials and vacant interstitials is allowed is 0.25. This explains the expression (2).

We further noticed that the fitting to the oxygen potential data on UO_2+0.5(_*_x-y_*_)_ did not require any extra entropy contributions. Thus, we assumed that the mixing of U^+4^/U^+5^ and U^+4^/U^+3^ does not produce independent contributions to the total configurational entropy. This could occur, if the electron distribution that differentiates U^+4^ from U^+5^ and U^+4^ from U^+3^ adjusts itself spontaneously to the distribution of interstitials or vacancies producing little of extra uncertainty. This assumption requires a further investigation; however, the current message is that a good fit to the oxygen potential data does suggest that the entropy effects from U^+4^/U^+5^ and U^+4^/U^+3^ are reduced.

1. **Derivation of expressions for the configurational entropy for U_1-_*_z_Ln_z_*O_2+0.5(_*_x-y_*_)_**

Modelling of UO_2_-*Ln*O_1.5_ mixtures requires taking into an account an additional entropy contribution from *Ln*/U mixing. When vacancies are present, this contribution is likely to be reduced relative to that of the ideal mixing entropy because cations necessarily occur in different local configurations. The average cation coordination number, <*K*>, is given by the equation <*K*> = 8 – 2*y*. The assumption of the absence of third-nearest vacancy-vacancy contacts excludes coordination numbers smaller than 7. Consequently, cations find themselves at least in two different local environments, 8-fold and 7-fold. The logical assumption is that the mixing of U and *Ln* occurs separately in 8- and 7-fold coordinated sites producing two independent contributions to the configurational entropy. The consistency with the relation <*K*> = 8 – 2*y* implies a certain relationship between fractions of 8-fold and 7-fold cations. It is easy to show that these fractions are 1– 2*y* and 2*y*, respectively. In a solid solution, in which the radii of III- and IV-valent cations have similar sizes, the III-valent cations are expected to be associated with vacancies [3]. This association implies that III valent and IV-valent cations partition differently into 7-fold and 8-fold sites. This partitioning is modelled here by requesting the coordination number of III-valent cations to be preferably 7. The study considers three different solutions for U and *Ln* partitioning between 7- and 8-fold sites, which are denoted as I, IIa and IIb. The separation of the entropy in two contributions and the partitioning of *Ln* into the 7-fold sites both cause a reduction in the computed value of the configurational entropy.

A special case of a hypo-stoichiometric solid solution occurs when U^+3^ and U^+5^ cations are absent and the fraction of vacancies is solely determined by the fraction of *Ln*O_1.5_ endmember. This condition corresponds to *y* = *z*. Thus, if all *Ln* atoms are 7-fold coordinated, the fraction of 7-fold *Ln* cations is *y* = *z*. As the total fraction of 7-fold cations must be 2*y* = 2*z*, a fraction *y* = *z* of U^+4^ cations must be transferred into the 7-fold coordination. The structural formula for this special case becomes $U_{1-2z}^{4,8}U_{z}^{4,7}{Ln}_{z}^{3,7}O_{2-0.5z}$.

**Hypo-stoichiometric** $\mathbf{U}_{\boldsymbol{1-2}\boldsymbol{y}}^{\boldsymbol{4,8}}\mathbf{U}_{\boldsymbol{y}}^{\boldsymbol{4,7}}\boldsymbol{Ln}_{\boldsymbol{z}}^{\boldsymbol{3,7}}\mathbf{U}_{\boldsymbol{y-z}}^{\boldsymbol{3,7}}\mathbf{O}_{\boldsymbol{2-0.5}\boldsymbol{y}}$ **solid solution, *z* < *y* < 0.5, type I**

The case when a fraction of U^+4^ is reduced to U^+3^ implies *y* > *z*, i.e. the fraction of vacancies is larger than the fraction, which can be created due to the sole presence of *Ln* cations. The additional fraction of 0.5(*y* – *z*) vacancies is thus solely due to the UO_1.5_ component. Thus,

the structural formula becomes$U_{1-2y}^{4,8}U_{y}^{4,7}{Ln}_{z}^{3,7}U_{y-z}^{3,7}O_{2-0.5y}$. This, formula, in turn, implies that *Ln*^+3^ can mix with U cations only over 2*y* 7-fold sites. Noting that the total fraction of 7-fold U atoms is 2*y* – *z*, the configurational entropy is given by the following equation

$S_{hypo,I}^{\mathrm{conf}}=-2yR(\frac{z}{2y}\ln\frac{z}{2y}+\frac{2y-z}{2y}\ln\frac{2y-z}{2y})$, (3)

which can be rewritten as follows

$S_{hypo,I}^{\mathrm{conf}}=-R(z\ln\frac{z}{2y}+\left( 2y-z \right)\ln\frac{2y-z}{2y})$. (4)

Note that the fraction 1 – 2*y* of $U^{4,8}$cations does not mix with *Ln* cations thus causing a decrease in the configurational entropy.

**Hypo-stoichiometric** $U_{1-2z}^{4,8}{Ln}_{z-y}^{3,8}U_{z-y}^{5,8}{Ln}_{y}^{3,7}U_{y}^{4,7}O_{2-0.5y}$ **solid solution, 0 < *y* < *z*, *z* < 0.5, type IIa**

This type of the solid solution corresponds to the case when all U^+3^ cations are oxidized to U^+4^, while a fraction of U^+4^ is further oxidised to U^+5^, however, no interstitials yet occur. Two special cases *z* < 0.5 and *z* > 0.5 can be distinguished. We consider the first one first.

The growth of the fraction of U^+5^ implies increasing the fraction of the stoichiometric U_0.5_*Ln*_0.5_O_2_ endmember. The fraction of vacancies decreases, and *y* parameter becomes smaller than *z*. Clearly, when all vacancies disappear (*y* = 0), all *Ln* atoms should contribute to the U_0.5_*Ln*_0.5_O_2_ endmember, while when the fraction of U^+5^ is zero, all *Ln* cations belong to *Ln*O_1.5_. Thus, the only solution for the structural formula is $U_{1-y-2(z-y)}^{4}{Ln}_{y}^{3}{Ln}_{z-y}^{3}U_{z-y}^{5}O_{2-0.5y}.$ However, we remember that the cations should be split into the fractions 2*y* of 1– 2*y* of 7-fold and 8-fold species, respectively. Logically, *Ln* cations that contribute to the stoichiometric endmember U_0.5_*Ln*_0.5_O_2_ should occur in the 8-fold coordination. Thus, the fraction of *Ln* in 7-fold coordination is *y*. This implies that additionally a fraction of *y* of U^+4^ cations should be transferred into the 7-fold coordination. The structural formula becomes $U_{1-2y-2(z-y)}^{4,8}{Ln}_{z-y}^{3,8}U_{z-y}^{5,8}{Ln}_{y}^{3,7}U_{y}^{4,7}O_{2-0.5y}$. This formula implies that a fraction *z* – *y* of 8-fold *Ln* cations can mix with 8-fold U atoms, while the fraction *y* of 7-fold *Ln* cations can separately mix with 7-fold U cations. Thus, the configurational entropy of cations has two contributions. Noting that the total fraction of 8-fold cations is 1 – 2*y*, and the total fraction of 8-fold U cations is $1-y-z$, the contribution of 8-fold cations is

$S_{hypo,IIa(8-fold)}^{\mathrm{conf}}=(1-2y)R(\frac{1-y-z}{1-2y}\ln\frac{1-y-z}{1-2y}+\frac{z-y}{1-2y}\ln\frac{z-y}{1-2y})$ , (5)

while the contribution of 7-fold cations is

$S_{hypo,IIa(7-fold)}^{\mathrm{conf}}=2yR(\frac{y}{2y}\ln\frac{y}{2y}+\frac{y}{1-2y}\ln\frac{y}{2y})$ . (6)

Both contributions are combined below in one equation as follows

$S_{hypo,IIa}^{\mathrm{conf}}=2Ry\ln2-R((1-y-z)\ln\frac{1-y-z}{1-2y}+\left( z-y \right)\ln\frac{z-y}{1-2y})$ . (7)

The structural formula $U_{1-2y-2(z-y)}^{4,8}{Ln}_{z-y}^{3,8}U_{z-y}^{5,8}{Ln}_{y}^{3,7}U_{y}^{4,7}O_{2-0.5y}$ can be rewritten as $U_{1-2z}^{4,8}{Ln}_{z-y}^{3,8}U_{z-y}^{5,8}{Ln}_{y}^{3,7}U_{y}^{4,7}O_{2-0.5y}$, from which it becomes clear that the model IIa is valid only at *z* < 0.5.

**Hypo-stoichiometric** $\mathbf{U}_{\boldsymbol{1-2}\boldsymbol{z-y}}^{\boldsymbol{4,7}}\boldsymbol{Ln}_{\boldsymbol{y+z-0.5}}^{\boldsymbol{3,7}}\mathbf{U}_{\boldsymbol{z-0.5}}^{\boldsymbol{5,7}}\boldsymbol{Ln}_{\boldsymbol{0.5-y}}^{\boldsymbol{3,8}}\mathbf{U}_{\boldsymbol{0.5-y}}^{\boldsymbol{5,8}}\mathbf{O}_{\boldsymbol{2-0.5}\boldsymbol{y}}$ **solid solution, 2*z* –** **1 < *y* < 0.5, 0.5 < *z* < 0.75, type IIb**

At *z* = 0.5 the structural formula of the model IIa can be written ${Ln}_{0.5-y}^{3,8}U_{0.5-y}^{5,8}{Ln}_{y}^{3,7}U_{y}^{4,7}O_{2-0.5y}$, implying that the condition of 1 – 2*y* for the total fraction of 8-fold cations is satisfied as a limiting case. A possible solution for the case of *z* > 0.5 is that the fractions of ${Ln}^{3,8}$ and $U^{5,8}$ are both kept equal to 0.5 – *y*, while the rest amount of ${Ln}^{3,8}$ cations of *z* – 0.5 and the same amount of $U^{5,8}$are transformed into the 7-ford configuration. The total fraction of ${Ln}^{3,7}$ is then *y* + *z* – 0.5. The fraction of $U^{4,7}$is then calculated as 1 – *z* – (0.5 – *y* + *z* – 0.5) = 1 – 2*z* + *y*. The structural formula takes the form $U_{1-2z-y}^{4,7}{Ln}_{y+z-0.5}^{3,7}U_{z-0.5}^{5,7}{Ln}_{0.5-y}^{3,8}U_{0.5-y}^{5,8}O_{2-0.5y}$. The configurational entropy is again composed of two contributions

$S_{hypo,IIb(7-fold)}^{\mathrm{conf}}=-2yR(\frac{y+ z-0.5}{2y}\ln\frac{y+ z-0.5}{2y}+\frac{y-z+0.5}{2y}\ln\frac{y-z+0.5}{2y})$ and (8)

$S_{hypo,IIb(8-fold)}^{\mathrm{conf}}=-(1-2y)R(\frac{0.5-y}{1-2y}\ln\frac{0.5-y}{1-2y}+\frac{0.5-y}{1-2y}\ln\frac{0.5-y}{1-2y})$ , (9)

which together give the equation

$S_{hypo,IIb}^{\mathrm{conf}}=-2R(0.5-y)\ln\frac{0.5-y}{1-2y}-R((y+z-0.5)\ln\frac{y+ z-0.5}{2y}+\left( y-z+0.5 \right)\ln\frac{y-z+0.5}{2y})$ . (10)

The model IIb is valid under the condition of $U^{4,7}$> 0, which is equivalent to *y* > 2*z* – 1. Clearly, stoichiometric states cannot occur if *z* > 0.5. As *y* is constrained to be less than 0.5, *z* value cannot be larger than 0.75.

**Hyper-stoichiometric solution** $\mathbf{U}_{\boldsymbol{1-x-2}\boldsymbol{z}}^{\boldsymbol{4,8}}\boldsymbol{Ln}_{\boldsymbol{z}}^{\boldsymbol{3,8}}\mathbf{U}_{\boldsymbol{z}}^{\boldsymbol{5,8}}\mathbf{U}_{\boldsymbol{x}}^{\boldsymbol{5,9}}\mathbf{O}_{\boldsymbol{2+0.5}\boldsymbol{x}}$**, type III**

At hyper-stoichiometric compositions vacancies are absent. Thus, the fraction *z* of *Ln*^+3^ cations (i.e. all *Ln* cations) and the same fraction *z* of U^+5^ cations are assumed to be in 8-fold coordination. The rest U^+5^ cations that balance the interstitials are formally prescribed coordination number of 9. As one interstitial occurs per one atom of metal, the fraction of $U^{5,9}$ is *x*. The structural formula of doped hyper-stoichiometric solid solution is then$U_{1-x-2z}^{4,8}{Ln}_{z}^{3,8}U_{z}^{5,8}U_{x}^{5,9}O_{2+0.5x}$. This formula implies that the fraction *z* of *Ln*^+3^ cations can be mixed with 8-fold U cations over the fraction 1 – *x* of 8-fold coordinated sites. The configurational entropy of cations is given by the equation

$S_{\mathrm{hyper}}^{\mathrm{conf}}=-(1-x)R(\frac{1-x-z}{1-x}\ln\frac{1-x-z}{1-x}+\frac{z}{1-x}\ln\frac{z}{1-x})$ , (11)

which can be rewritten as follows

$S_{\mathrm{hyper}}^{\mathrm{conf}}=-R((1-x-z)\ln\frac{1-x-z}{1-x}+z\ln\frac{z}{1-x})$ . (12)

1. **Computational details**

The free energy of each solid solution type is combined of the enthalpy and entropy terms as shown in the main text. Generally, the free energy is a function of the composition, *z*, the temperature, *T*, the oxygen partial pressure and *y* or *x* parameter, depending on the solid solution type. A simple FORTRAN code varies the $\log({P_{O_{2}}}/{P^{0}})$ parameter within the limits of –40 to 0 at fixed values of *z* and *T*. At each set of *z,* *T* and $\log({P_{O_{2}}}/{P^{0}})$ the free energy is minimised with respect to either *y* or *x*. The same calculations are performed for each of the four models, i.e. I, IIa, IIb and III. The model that gives the lowest free energy value for the given set of *z,* *T* and $\log({P_{O_{2}}}/{P^{0}})$ is chosen to represent equilibrium and is used to plot $\log({P_{O_{2}}}/{P^{0}})$ as a function of 0.5(*x* – *y*). The theoretical function is plotted together with the experimental data. The values of enthalpic and entropic parameter of the endmembers and the Margules parameters are manually adjusted until the agreement between the theory and experiment is considered satisfactory. The parameters of *Ln*-free system are determined first and are kept fixed in all UO_2_ – *Ln*O_1.5_ systems.

1. Bukaemskiy A. A., Vinograd V. L., Kowalski P. M. Ion distribution models for defect fluorite ZrO_2_ - AO_1.5_ (A = Ln, Y) solid solutions: I. Relationship between lattice parameter and composition. Acta Mater. 2021;202:99-111. doi: <https://doi.org/10.1016/j.actamat.2020.10.045>

2. Vinograd V. L., Bukaemskiy A. A. Ion distribution models for defect fluorite ZrO_2_ - AO_1.5_ (A = Ln, Y) solid solutions: II. Thermodynamics of mixing and ordering. Acta Mater. 2021;202:55-67. doi: <https://doi.org/10.1016/j.actamat.2020.10.046>

3. Solomon J. M., Alexandrov V., Sadigh B., Navrotsky A., Asta M. Computational study of the energetics and defect clustering tendencies for Y- and La-doped UO_2_. Acta Mater. 2014;78:282-9. doi: <https://doi.org/10.1016/j.actamat.2014.06.052>
